# Assessment of Gram- and Viability-Staining Methods for Quantifying Bacterial Community Dynamics Using Flow Cytometry

**DOI:** 10.3389/fmicb.2020.01469

**Published:** 2020-06-26

**Authors:** Aurore Duquenoy, Samuel Bellais, Cyrielle Gasc, Carole Schwintner, Joël Dore, Vincent Thomas

**Affiliations:** ^1^MaaT Pharma, Lyon, France; ^2^Bioaster, Institut de Recherche Technologique, Paris, France; ^3^Université Paris-Saclay, INRAE, MetaGenoPolis, AgroParisTech, MICALIS, Jouy-en-Josas, France

**Keywords:** flow cytometry population dynamics, anaerobic sorting, gut microbiota, *in vitro* fermentation, Gram staining, Live/Dead staining

## Abstract

Over the past years, gut microbiota became a major field of interest with increasing reports suggesting its association with a large number of human diseases. In this context, there is a major interest to develop analysis tools allowing simple and cost-effective population pattern analysis of these complex ecosystems to follow changes over time. Whereas sequence-based metagenomics profiling is widely used for microbial ecosystems characterization, it still requires time and specific expertise for analysis. Flow cytometry overcomes these disadvantages, providing key information on communities within hours. In addition, it can potentially be used to select, isolate and cultivate specific bacteria of interest. In this study, we evaluated the culturability of strictly anaerobic bacteria that were stained with a classical Live/Dead staining, and then sorted using flow cytometry under anaerobic conditions. This sorting of “viable” fraction demonstrated that 10–80% of identified “viable” cells of pure cultures of strictly anaerobic bacteria were culturable. In addition, we tested the use of a combination of labeled vancomycin and Wheat Germ Agglutinin (WGA) lectin to discriminate Gram-positive from Gram-negative bacteria in complex ecosystems. After validation on both aerobic/anaerobic facultative and strictly anaerobic bacteria, the staining methods were applied on complex ecosystems, revealing differences between culture conditions and demonstrating that minor pH variations have strong impacts on microbial community structure, which was confirmed by 16S rRNA gene sequencing. This combination of staining methods makes it possible to follow-up evolutions of complex microbial communities, supporting its future use as a rapid analysis tool in various applications. The flow cytometry staining method that was developed has the potential to facilitate the analysis of complex ecosystems by highlighting changes in bacterial communities’ dynamics. It is assumed to be applicable as an efficient and fast approach to improve the control of processes linked to a wide range of ecosystems or known communities of bacterial species in both research and industrial contexts.

## Introduction

Human gut microbiota are composed of a wide variety of microorganisms including bacteria, archaea, fungi, viruses and yeasts (Lozupone et al., 2012; Dieterich et al., 2018). Bacteria are the most studied microorganisms with around 10^11^ bacteria per gram of stool and a predominance of Firmicutes and Bacteroidetes (Qin et al., 2010). The analysis of gut microbiota led to the concept of enterotypes that divide human microbiota based on the enrichment of specific taxa (Arumugam et al., 2011; Wu et al., 2011). Diverse and specific to each individual, gut microbiota can be influenced by different factors such as age, sex, diseases, drug treatments, environment or nutrition (Lozupone et al., 2012). A range of disorders was also shown to be associated with altered gut microbiota composition (Lin and Zhang, 2017). Based on these observations, the concept of Next Generation Probiotics (NGP) gained interest. It relies on the use of bacterial species belonging to commensal genera such as *Akkermansia*, *Christensenella* or butyrate-producers *Faecalibacterium*, *Roseburia* and *Eubacterium* to restore gut microbiota equilibrium. These species showed potential interest owing to anti-inflammatory properties (Chang et al., 2019). Another strategy to modulate or correct dysbiotic microbiota involves Fecal Microbiota Transplantation (FMT) that was shown to be highly effective in the treatment of recurrent *Clostridioides difficile* infections (Quraishi et al., 2017; Ianiro et al., 2018). Meanwhile, a large number of trials are ongoing, exploring other potential therapeutic applications (Allegretti et al., 2019).

The responses of complex microbial communities to various factors, including the environment, life style, dietary habits, food supplements or drug treatments are often investigated using *in vitro* fermentation systems (Geirnaert et al., 2017) consisting of several reactors that mimic different parts of the gastrointestinal (GI) tract (Williams et al., 2015). Molecular methods based on qPCR or Next Generation Sequencing (NGS) are generally used to analyze gut microbiota compositions. Having the advantage of detecting “unculturable” micro-organisms, the usual NGS technologies present specific drawbacks such as the need to wait for several days or even weeks before obtaining reports if sequencing is outsourced or performed by a platform, or the lack of metabolic activity monitoring. This is an important limitation for daily follow-up of fermentation or for the investigation/optimization of culture parameters in experimental and industrial contexts. On the other hand, the great potential of cytometry as a tool for rapid analysis of the intestinal microbiota has been reported since its development in the 1990s (van der Waaij et al., 1994). It has been frequently used in combination with fluorescently labeled 16s rRNA targeting probes, referred as Flow-FISH (Fluorescent *In Situ* Hybridization) technique, for compositional analysis of intestinal microbiota (Thomas et al., 2002; Mueller et al., 2006). This analytical tool has been increasingly used in recent years for the analysis of complex ecosystems and also as a complementary tool to sequencing to pre-select samples by examining microbial community concentrations or monitoring undesirable bacterial overgrowth (Prest et al., 2014). The forward-scattered count (FSC) and the side-scattered count (SSC) signals depend on physical properties of analyzed particles and can be combined with auto-fluorescence measurement to assess microbial communities’ changes without any staining (Dhoble et al., 2016). However, staining of samples offers a multi-parametric analysis providing deeper information on the studied community (Buysschaert et al., 2016). In the context of bacterial analysis, nucleic acid stains are mainly used as well as lectins, labeled antibiotics or specific dyes to evaluate membrane potential or metabolic activities (Muller and Nebe-von-Caron, 2010). 4’,6-diamidino-2’-phenylindole (DAPI), a fluorescent dye that binds to A-T rich sequences of double stranded DNA, has been used in conjunction with FCM in many applications and enabled to identify specific subgroups of complex communities in environmental samples (Koch et al., 2013), gut microbiota (Zimmermann et al., 2016) or salivary microbiota (van Gelder et al., 2018). More recently, Krause et al. (2020) used it to follow the dynamics of a simplified human microbiota composed of eight bacterial species in a fermenter. However, the use of DAPI requires a UV laser for excitation, which is costly and thus less frequently available than other lasers. Thus, we were interested in investigating complementary staining methods that involve the use of more affordable lasers commonly found on flow cytometers. Other nucleic acids stains including Syto 9, SYBR Green I as well as Syto 24 and propidium iodide (PI) are often used to assess bacterial viability (Berney et al., 2007; Nescerecka et al., 2016; Chiron et al., 2018). The ready to use Live/Dead^®^ BacLight^TM^ bacterial viability kit combining Syto 9 and PI stains allowed to rapidly evaluate bacterial viability. However, misleading results were previously obtained with PI staining (Stiefel et al., 2015). Other strategies that use the combination of nucleic acid stains Syto13 and hexidium iodide (HI) were also demonstrated to identify Gram-positive bacteria within mixes of pure cultures (Mason et al., 1998). Gram-staining techniques have also been described through the use of Wheat Germ Agglutinin (WGA), a lectin that binds to specific carbohydrates (De Hoff et al., 2009) including *N*-acetylglucosamine and *N*-acetylneuraminic acid residues present in the peptidoglycan layer (Wittmann and Pieters, 2013). The WGA lectin was also used to identify Gram-positive bacteria in mixed cultures of known bacteria (Ruger et al., 2012). Labeled vancomycin was also previously used for similar kind of applications, including the analysis of Gram-positive bacteria from mouse gut microbiota using microscopy (Wang et al., 2017). This antibiotic has the ability to inhibit cell wall synthesis by binding to C-terminal D-Alanine-D-Alanine residues of the peptidoglycan precursors (van Hoek et al., 2011) but the molecule is too large to penetrate the outer membrane of the Gram-negative bacteria (Fernandes et al., 2017).

In order to determine staining procedures that would be applicable to a range of microbial environments, which was a specific need expressed during the CYTO 2018 Conference Workshops (Czechowska et al., 2019), we investigated the use of a specific staining method in combination with FCM to rapidly characterize and monitor complex ecosystems obtained by *in vitro* fermentation. We focused on Gram-staining with the combination of labeled WGA and vancomycin and the evaluation of Live/Dead proportions of bacteria. The methods were developed and validated with pure cultures of aerobic and facultative or strictly anaerobic bacteria before being used on complex ecosystems.

## Materials and Methods

### Ethics

The fecal material was collected anonymously by the company MaaT Pharma from healthy volunteers. In compliance with the Article L1243-3 of the French Health Code, documents describing the collection were filed under the ref DC-2016-2609. Donors were informed that no personal information and no analysis of the human material would be performed and, as a consequence, that no information could be delivered concerning the use of their donation. The file has been accepted by the French authorities the 15th May 2016.

### Bacterial Strains and Culture Media

A total of 54 strains including 40 aerobic/facultative anaerobic strains and 14 strictly anaerobic strains corresponding in total to 20 species, were used for the experiments. Aerobic strains were cultured at 37°C under 180 rpm agitation. Anaerobic commensal strains were grown at 37°C using an anaerobic chamber (BACTRON 600 – Sheldon) filled with an atmosphere of 90% N_2_ + 5% H_2_ + 5% CO_2_. Anaerobic atmosphere was verified with resazurin color indicators (BR0055 – Oxoid). Culture media, PBS and all other materials used for cultivation were placed in the chamber at least 48 h before use to reduce to anaerobic conditions.

Brain heart Infusion (BHI) and Luria Berthani (LB) media were used for Gram-positive and Gram-negative aerobic/facultative anaerobic strains. The modified Gifu Anaerobic Medium (mGAM, Hyserve) was used for strictly anaerobic strains and was supplemented either with 30% bovine rumen (mGAMr) or 0.1% cellobiose, 0.1% inulin, 0.25% mucin and 30% bovine rumen (mGAM CRIM). All strains and their corresponding culture media are presented in Tables 1, 2.

**TABLE 1:** Aerobic/facultative anaerobic bacteria strains, culture media and Gram status.

**Designation**	**Phylum**	**Family**	**Strains**	**Origin**	**Culture medium**	**Gram**
*Bacillus subtilis*	Firmicutes	*Bacillaceae*	ATCC^®^ 23857^TM^	Unknown	BHI	+
*Bacillus subtilis*	Firmicutes	*Bacillaceae*	ATCC^®^ 6633^TM^	Unknown	BHI	+
*Bacillus subtilis*	Firmicutes	*Bacillaceae*	OxyR	Unknown	BHI	+
*Enterococcus faecalis*	Firmicutes	*Enterococcaceae*	ATCC^®^27959^TM^	Bovine udder infection	BHI	+
*Enterococcus faecalis*	Firmicutes	*Enterococcaceae*	ATCC^®^ 29212^TM^	Urine	BHI	+
*Enterococcus faecalis*	Firmicutes	*Enterococcaceae*	ATCC^®^ 29302^TM^	Unknown	BHI	+
*Enterococcus faecalis*	Firmicutes	*Enterococcaceae*	ATCC^®^ 33012^TM^	Unknown	BHI	+
*Enterococcus faecalis*	Firmicutes	*Enterococcaceae*	ATCC^®^ 33186^TM^	Unknown	BHI	+
*Enterococcus faecalis*	Firmicutes	*Enterococcaceae*	ATCC^®^ 49477^TM^	Clinical isolate	BHI	+
*Enterococcus faecalis*	Firmicutes	*Enterococcaceae*	ATCC^®^ 51188^TM^	Clinical isolate	BHI	+
*Enterococcus faecalis*	Firmicutes	*Enterococcaceae*	ATCC^®^ 51299^TM^	Peritoneal fluid	BHI	+
*Enterococcus faecalis*	Firmicutes	*Enterococcaceae*	ATCC^®^ 700802^TM^	Human blood from patient	BHI	+
*Enterococcus faecalis*	Firmicutes	*Enterococcaceae*	JH2.2	Unknown	BHI	+
*Enterococcus faecalis*	Firmicutes	*Enterococcaceae*	ATCC^®^ 19433^TM^	Unknown	BHI	+
*Escherichia coli*	Proteobacteria	*Enterobacteriaceae*	ATCC^®^ 25922^TM^	Clinical isolate	LB	-
*Escherichia coli*	Proteobacteria	*Enterobacteriaceae*	ATCC^®^ 35218^TM^	Canine	LB	-
*Escherichia coli*	Proteobacteria	*Enterobacteriaceae*	CIP 54.127	Unknown	LB	-
*Klebsiella pneumoniae*	Proteobacteria	*Enterobacteriaceae*	ATCC^®^ 700603^TM^	Urine from hospitalized patient	LB	-
*Klebsiella pneumoniae*	Proteobacteria	*Enterobacteriaceae*	ATCC^®^ 10031^TM^	Unknown	LB	-
*Klebsiella pneumoniae*	Proteobacteria	*Enterobacteriaceae*	NTUH-K2044	Taiwanese patient with liver abscess and meningitis	LB	-
*Lactococcus lactis* subsp. *lactis*	Firmicutes	*Streptococcaceae*	ATCC^®^ 11454^TM^	Unknown	BHI	+
*Lactococcus lactis* subsp. *cremoris*	Firmicutes	*Streptococcaceae*	MG1363	Unknown	BHI	+
*Salmonella enterica* Ser. Typhimurium	Proteobacteria	*Enterobacteriaceae*	ATCC^®^13311^TM^	Human feces	LB	-
*Salmonella enterica* Ser. Agona	Proteobacteria	*Enterobacteriaceae*	Se2	Food	LB	-
*Salmonella enterica* Ser. Brandenburg	Proteobacteria	*Enterobacteriaceae*	Se3	Food	LB	-
*Salmonella enterica* Ser. Dublin	Proteobacteria	*Enterobacteriaceae*	Se5	Food	LB	-
*Salmonella enterica* Ser. Duby	Proteobacteria	*Enterobacteriaceae*	Se4	Food	LB	-
*Salmonella enterica* Ser. Enteritidis	Proteobacteria	*Enterobacteriaceae*	Se6	Food	LB	-
*Salmonella enterica* Ser. Hadar	Proteobacteria	*Enterobacteriaceae*	Se7	Food	LB	-
*Salmonella enterica* Ser. Indiana	Proteobacteria	*Enterobacteriaceae*	Se8	Food	LB	-
*Salmonella enterica* Ser. Saint-Paul	Proteobacteria	*Enterobacteriaceae*	Se1	Food	LB	-
*Staphylococcus aureus*	Firmicutes	*Staphylococcaceae*	ATCC^®^ 13301^TM^	Unknown	BHI	+
*Staphylococcus aureus*	Firmicutes	*Staphylococcaceae*	ATCC^®^ 27659^TM^	Double-mutant derived from J. Baldwin strain U9	BHI	+
*Staphylococcus aureus*	Firmicutes	*Staphylococcaceae*	ATCC^®^ 25923^TM^	Clinical isolate	BHI	+
*Staphylococcus aureus*	Firmicutes	*Staphylococcaceae*	ATCC^®^ 27217^TM^	Nares of nurse	BHI	+
*Staphylococcus aureus*	Firmicutes	*Staphylococcaceae*	ATCC^®^ 27661^TM^	Unknown	BHI	+
*Staphylococcus aureus*	Firmicutes	*Staphylococcaceae*	ATCC^®^ 29213^TM^	Wound	BHI	+
*Staphylococcus aureus*	Firmicutes	*Staphylococcaceae*	ATCC^®^ 29247^TM^	Unknown	BHI	+
*Staphylococcus aureus*	Firmicutes	*Staphylococcaceae*	ATCC^®^ 8096^TM^	Furuncle	BHI	+
*Staphylococcus aureus*	Firmicutes	*Staphylococcaceae*	CIP 4.83	Human lesion	BHI	+

*Ser, Serovars; BHI, Brain Heart Infusion; LB, Luria Berthani.*

**TABLE 2:** Strictly anaerobic bacteria strains, culture media and Gram status.

**Designation**	**Phylum**	**Family**	**Strains**	**Origin**	**Culture medium**	**Gram**
*Akkermansia muciniphila*	Verrucomicrobia	*Akkermansiaceae*	DSM 22959	Human intestinal tract (feces)	mGAMr/mGAM CRIM	−
*Alistipes finegoldii*	Bacteroidetes	*Rikenellaceae*	DSM 17242	Human, appendix tissue	mGAMr	+
*Bacteroides fragilis*	Bacteroidetes	*Bacteroidaceae*	DSM 2151	Appendix abscess	mGAMr	−
*Bacteroides thetaiotaomicron*	Bacteroidetes	*Bacteroidaceae*	DSM 2079	Human feces	mGAMr	–
*Christensenella minuta*	Firmicutes	*Christensenellaceae*	DSM 22607	Feces from healthy Japanese male	mGAMr/mGAM CRIM	−
*Clostridium scindens*	Firmicutes	*Lachnospiraceae*	DSM 5676	Human feces	mGAMr	+
*Eubacterium hallii*	Firmicutes	*Lachnospiraceae*	DSM 3353	Human feces	mGAMr	+
*Eubacterium hallii*	Firmicutes	*Lachnospiraceae*	DSM 17630	Infant, fecal sample	mGAMr	+
*Prevotella copri*	Bacteroidetes	*Prevotellaceae*	DSM 18205	Human feces	mGAMr	−
*Roseburia intestinalis*	Firmicutes	*Lachnospiraceae*	DSM 14610	Infant feces	mGAMr	−/+
*Subdoligranulum variabile*	Firmicutes	*Ruminococcaceae*	DSM 15176	Human feces	mGAMr	−/+
*[Ruminococcus]_torques*	Firmicutes	*Lachnospiraceae*	PCK 18	Chicken caecum	mGAM	+
*[Clostridium]_spiroforme_JCM_1432*	Firmicutes	*Erysipelotrichaceae*	PCK 31	Chicken caecum	mGAM	+
*Flavonifractor_plautii_aK2*	Firmicutes	*Ruminococcaceae*	PCK 48	Chicken caecum	mGAM	−/+

*mGAMr: modified Gifu Anaerobic Medium + 30% bovine rumen, mGAM CRIM: mGAM + 30% bovine rumen + 0.1% cellobiose + 0.1% inulin + 0.25% mucin.*

### Labeling Stains

The lectin Alexa-Fluor 647^®^ Wheat Germ Agglutinin (WGA-647 – Invitrogen – W32466) was suspended in sterile distilled water to obtain a stock solution at 1 mg/mL. A stock solution of Bodipy-FL Vancomycin (Van-Bodipy, Invitrogen – V34850) was prepared in DMSO at 0.5 mg/mL. Aliquotes of both stock solutions were stored at -20°C. For Gram analysis, the LIVE BacLight^TM^ Bacterial Gram Stain Kit (Invitrogen – L7005) was also used as recommended by the manufacturer. This kit is composed of Syto 9 and HI at 3.34 and 4.67 mM, respectively. For viability analysis, the Live/Dead^®^ BacLight^TM^ bacterial viability kit (Invitrogen – L34856) was used as recommended by the manufacturer. This kit is composed of Syto 9 (3.34 mM in DMSO) and propidium iodide (PI) (20 mM in DMSO), two nucleic acid stains that stain all bacteria and in preference bacteria with a damaged cell wall that are considered as “dead,” respectively (Berney et al., 2007).

### Flow Cytometry Analysis

The Influx^®^ cell sorter (BD) was used for cytometry analysis. It is equipped with a 488 nm laser (power of 200 mW), 405 nm laser (Power of 100 mW) and 640 laser (Power of 120 mW) to obtain Forward (FSC) and Side scatter (SSC) signals at 488 nm. Fluorescence of Syto 9, PI and HI were excited with the 488 nm laser and collected through 540/30 nm band pass (BP) filter for Syto 9 and 670/30 nm BP for both PI and HI. No compensation was applied when using Syto 9 and PI or Syto 9 and HI because of the low or even absent spillover of fluorescence emitted in the band pass used. Fluorescence of Van-Bodipy was excited with the 488 nm laser and collected through the 540/30 nm BP. WGA-647 fluorescence was excited with the 640 nm laser and collected through the 670/30 nm BP. Using two different lasers for excitation of Van-Bodipy and WGA-647, no compensation was applied when using both stains. Before running samples, adjustment of area scaling and quality control were performed using rainbow beads (Invitrogen – L34856) and the flow rate was maintained around 100–150 events/second. Analysis of the samples was carried out with a flow rate around 500–2,000 events/seconds. The total number of recorded particles was determined at 50,000 events.

Data analysis and gating were performed using the FlowJo software (FlowJo LLC, Ashland, OR, United States). Bacterial quantification was performed using microsphere standard beads (Invitrogen – L34856). Background noise was removed by considering only events with FSC values above 10^1^. To discriminate cells from background in the viability analysis, a gate was applied for bacteria detection. Only events with a Syto 9 positive fluorescence were referred to bacteria (Supplementary Figure S1). For determination of viability, gates were set based on an isopropanol-treated control (Supplementary Figure S1). The viability was calculated as the number of “viable” cells over the total number of cells (“viable” and “non-viable” cells). For Gram proportion, manual gating was performed around visible groups of populations.

### Optimization of Gram Staining in Flow Cytometry

Overnight cultures of *Bacillus subtilis* ATCC 23857, *Enterococcus faecalis* ATCC 29212, *Enterococcus faecalis* JH2.2, *Lactococcus lactis* ATCC 11454, *Klebsiella pneumoniae* ATCC 700603, *Salmonella enterica typhimurium* ATCC 13311 and *Escherichia coli* ATCC 35218, were adjusted to an optical density (OD_600_) of 0.5 based on spectrophotometer reading (Ultrospec 10 cell Density) corresponding to 1.0 × 10^8^ to 1.0 × 10^9^ cells per ml depending on the species. Bacteria were either exposed to 1) 0.1; 0.2; 0.5; 1; 2 or 4 μg/mL of Van-Bodipy, 2) equal mixture of Van-Bodipy and unlabeled vancomycin (Van) at 1, 2, 4, and 8 μg/mL or 3) 10, 20, 30, 50, 60, 80, or 100 μg/mL of WGA-647 final concentrations for 15 min in the dark at room temperature. Combination of both stains Van-Bodipy/Van and WGA-647 were also investigated with their required concentrations with or without 1 M KCl that has been shown to improve WGA-647 staining (Holm and Jespersen, 2003). A washing step (centrifugation at 10,000 *g* for 5 min) was added to remove unbound stains. Samples were suspended in 200 μL of PBS and then analyzed using the Influx^®^ cell sorter as described above.

### Impact of Fixation on Gram Staining

Overnight cultures of Gram-positive and Gram-negative bacteria were adjusted to an OD_600_ of 0.5 based on spectrophotometer measurement. Cultures were either diluted in PBS or 1 M KCl and then exposed to 1% paraformaldehyde (PFA) or 95% ethanol during 1 h at room temperature. After two washing step in PBS, staining with both Van-Bodipy/Van/WGA-647 at their optimal concentration of 2/2/20 μg/mL was then performed either in 1 M KCl or PBS during 15 min, in the dark at room temperature. A washing step was performed before FCM analysis.

### Gram Screening of Aerobic/Facultative Anaerobic Strains and Strictly Anaerobic Bacteria

Overnight cultures were adjusted to an OD_600_ of 0.5 for aerobic/facultative anaerobic bacteria based on spectrophotometer measurement or in the range of 10^5^ to 10^7^ events/mL based on flow cytometry quantification for strictly anaerobic bacteria. A mixture of Van-Bodipy/Van/WGA-647 at their optimal concentrations of 2/2/20 μg/mL in 1 M KCl was used. After 15 min of staining at room temperature in the dark, bacteria were washed in PBS before FCM analysis. Staining was performed on three biological replicates.

Classical Gram staining with crystal violet and safranin was performed in parallel (77730 Gram Staining kit, Sigma-Aldrich) and the microscope slides were observed using the 100 X oil-immersion objective.

The commercially available LIVE BacLight^TM^ Bacterial Gram Stain Kit combining Syto 9 and HI was used as recommended by the manufacturer on overnight cultures of *E. coli* BL21, *E. faecalis* ATCC 29212 and *S. variabile* DSM 15146 adjusted in the range of 10^5^ to 10^7^ events/mL based on flow cytometry quantification.

### Evaluation of Live/Dead Staining, Anaerobic Sorting of Single Strains

To evaluate culturability of the “viable” and “non-viable” fractions, bacterial cells were sorted according to their Live/Dead staining status and then cultured in anaerobic conditions. To do so, the Influx^®^ cell sorter was modified as described before (Thompson et al., 2015). Briefly, a glove box was plugged on the sorting chamber, creating an airtight environment around the sort stream. Nitrogen was flushed inside the glove box to decrease oxygen concentration below 0.8% as indicated with the ToxiRAE Pro sensor (PGM-1860 – RAE systems).

The bacteria used for sorting experiments were anaerobically cultured for 24 or 48 h at 37°C on mGAMr plates. One colony was then sub-cultured in mGAMr broth or mGAM CRIM broth (*A. muciniphila* and *C. minuta*) for 24 h for up to 5 days. The culture time was determined depending on the growth curve kinetics of each tested strain to reach the stationary phase and obtain a mixture of Syto 9- and Syto 9/PI-stained cells enabling to perform the sorting of “viable” and “non-viable” fractions. Bacteria were adjusted in the range of 10^7^ to 10^8^ events/mL based on flow cytometry quantification and stained in reduced PBS (0.5 mg/L resazurin, 2.1 mM soldium sulfur and 2.8 mM L-cystein HCl) with Syto 9 and PI for 15 min in the dark at room temperature at 5 μM and 30 μM final concentration, respectively. Stained samples were then washed and resuspended in reduced PBS and covered with 500 μL of paraffin oil to prevent oxygen exposure before FCM analysis. Pre-reduced mGAMr agar medium prepared in Nunc OmniTray Single-Well Plates (Thermo Scientific) were sealed in a GenBag before being transferred from the anaerobic chamber to the cell sorter glove box. Sorting was performed on these plates with a 14 × 8 model consisting in 14 columns and 8 lines. Syto 9- and Syto 9/PI-stained bacteria were sorted in triplicate following the sorting pattern of 1, 3, 10, 30, 100, 300, and 1,000 events per spots with the one drop single function. The flow rate was adjusted to 1,000 events/seconds. At the end of the sorting, agar plates were sealed again in the GenBag before being transferred in the anaerobic chamber for 24–48 h of incubation at 37°C.

### Fecal Samples Collection and Fermentation Medium

Stool samples were collected anonymously from healthy human volunteers as presented in the “Ethics” section. Based on 16S rRNA gene repertoire sequencing, three fecal samples F1, F2, and F3 representing the three described enterotypes (Arumugam et al., 2011) were used for the fermentation experiments performed with the mGAM medium classically used to culture commensal anaerobic bacteria (Gotoh et al., 2017) and previously used in fermentation experiments (Takagi et al., 2016).

### Micro-24^®^ Microbioreactor Parameters and Sampling

Batch fermentation was performed in a 24-wells Micro-24^®^ cassette (MRT-PRC, Pall) using specific caps allowing to limit external gas exchanges. Culture medium and fecal samples F1, F2 and F3 were processed under the anaerobic chamber. After addition of 5 mL of the culture medium in each independent well of the cassette, the pH calibration was performed as described by the manufacturer. Briefly, an overnight incubation of the cassette with only culture medium was performed at 37°C under the corresponding gas supply to maintain anaerobic conditions. An offline measurement of the pH after the incubation was performed to calculate the offset to apply in the controlling software of the Micro-24^®^ Microbioreactor. 25 μL of each thawed fecal sample was then added to the corresponding wells. The Micro-24^®^ cassette was loaded on the Micro-24^®^ and the fermentation was started. The Micro-24^®^ software directly monitored the pH, temperature and gas supply. Each experiment was carried out at 37°C, under an orbital agitation of 650 rpm and under anaerobic condition that were maintained with a gas supply of a mixture of CO_2_/N_2_ (75/25). The pH was regulated at 6.2 or 6.5 with NH_3_ supply.

For each fecal sample, the fermentation conditions were performed in triplicate. At each sampling time (24, 48, and 72 h), 500 μL of samples were collected to evaluate the quantity, viability and Gram proportion by flow cytometry and perform sequencing analysis. Viability and Gram proportion analysis were performed as previously described, and quantification was performed using microsphere standard beads (Invitrogen).

### DNA Extraction, Sequencing and Bioinformatics Analysis

Fermentation samples collected at 24 and 48 h were selected for 16S rRNA gene sequencing. Genomic DNA was extracted by Eurofins Genomics from the samples using the NucleoSpin Soil kit (Macherey Nagel). A sequencing library targeting the V3-V4 region of the 16S rRNA gene was constructed for each sample using the MyTaq HS-Mix (Bioline) according to the manufacturer’s instructions. Libraries were then sequenced in paired-end (2 × 300 bp) MiSeq v3 (Illumina) runs. After amplicon merging using FLASH (Magoc and Salzberg, 2011), reads were quality filtered using Trimmomatic (Bolger et al., 2014). Amplicons were then clustered into OTUs with an identity threshold of 97%, and a taxonomical annotation was assigned to each OTU using VSEARCH (Rognes et al., 2016) and the Silva SSU database Release 128. To allow data comparison, the number of sequences was normalized to 60,000 amplicons per sample. The α- and β-diversity indexes were calculated using R Statistical Software (R Core Team, 2015, version 3.4.4^1^) using vegan and phyloseq packages. 20 subsamplings per sample were used to estimate α-diversity indexes (richness, Shannon, Inverse Simpson) variabilities. β-diversity Principal Coordinates Analysis (PCoA) were created using the Bray-Curtis dissimilarity measure. 16S rRNA gene sequencing was performed on triplicates except for two samples from fecal samples F2 and F3 obtained after 48 h of fermentation of the culture condition regulated at pH 6.5, for which analysis was performed on duplicates.

### Comparison of Gram-Positive and Gram-Negative Bacterial Proportions Measured by FCM With Proportions Estimated From 16S rRNA Repertoire Sequencing

We compared proportions of Gram-positive and Gram-negative bacteria detected in complex microbiota using FCM analysis with putative Gram assignation of each OTU at the genus level (Supplementary Table S1 for Gram assignment). Two different calculations were used for microbiome profiling: the classical relative abundancy approach that is described in section of 2.11 of this manuscript, and the quantitative microbiome approach described by Vandeputte et al. (2017). In this method, it is proposed to calculate a 16S rRNA gene copy-number-corrected sequencing depth divided by sample cell count to take into account variations in 16S rRNA gene-copy numbers between species and variations in microbial loads between samples. Mean operon numbers were retrieved from the rrndb database (Stoddard et al., 2015) for each OTU at the genus level, being approximated to the closest phylogenetic level when the genus was not in the database (Supplementary Table S1 for 16S rRNA gene-copy numbers used for each OTU).

### Statistical Analysis

All fermentation data including cytometry and sequencing are expressed as the mean ± standard deviation.

## Results

### Optimizing Van-Bodipy and WGA-647 Concentrations to Improve Gram Differentiation

**FIGURE 1:**
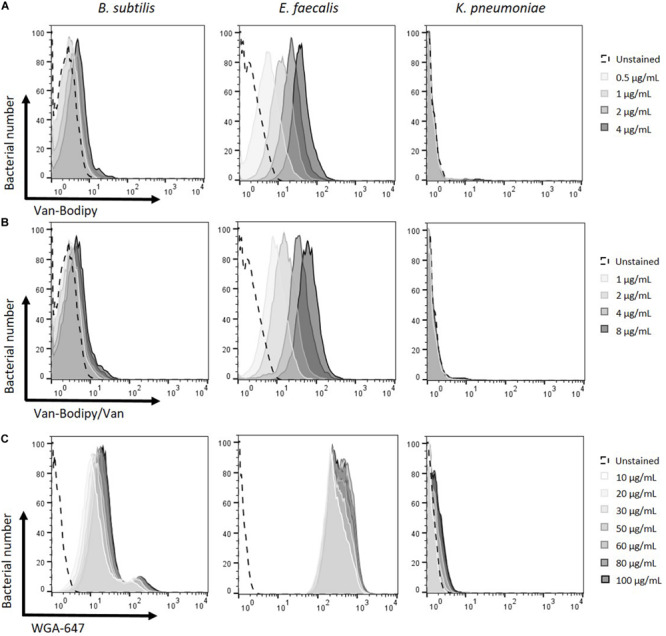
Optimization of fluorescent labeling for the discrimination of Gram-positive and Gram-negative bacteria. Different Van-Bodipy and WGA-647 concentrations were investigated. Overnight cultures of the tested bacteria *B. subtilis* ATCC 23857, *E. faecalis* JH2.2 and *K. pneumoniae* ATCC 700603 were adjusted at an OD_600_ of 0.5. The different stainings were performed for 15 min at room temperature with the different concentrations of **(A)** Van-Bodipy **(B)** Van-Bodipy/Van and **(C)** WGA-647. Higher concentrations are presented in darker grays **(A)** 0.5; 1; 2 or 4 μg/mL, **(B)** 1, 2, 4, and 8 μg/mL and **(C)** 10, 20, 30, 50, 60, 80, or 100 μg/mL final concentrations. Unstained bacterial species are presented in black dashed lines.

Different concentrations of both labeled stains Van-Bodipy and WGA-647 were investigated to determine the best staining condition to discriminate Gram-positive from Gram-negative bacteria. Overnight cultures of *B. subtilis* ATCC 23857, *E. faecalis* JH2.2 and *K. pneumoniae* ATCC 700603 adjusted to OD_600_ of 0.5 were used. Minor differences were observed on Gram-positive bacteria with concentrations of 0.1 and 0.2 μg/mL of Van-Bodipy (data not shown). Staining of *E. faecalis* JH2.2 was improved when progressively increasing concentrations of Van-Bodipy from 0.5 to 4 μg/mL with geometric mean values of Van-Bodipy fluorescence increasing from 5 to 38.5, whereas there was no significant improvement for *B. subtilis* ATCC 23857 for which geometric mean values of Van-Bodipy fluorescence were only slightly increased from 2.8 to 4.6 (Figure 1A). The Gram-negative species *K. pneumoniae* ATCC 700603 remained unstained by the Van-Bodipy, whatever the concentrations used, with geometric mean values of Van-Bodipy fluorescence around 1.1.

Mixtures of equal quantities of unlabeled vancomycin and Van-Bodipy were tested to improve the staining of *B. subtilis* as previously reported (Tiyanont et al., 2006). Final concentrations from 1 to 8 μg/mL of Van-Bodipy/Van were investigated. As previously observed with Van-Bodipy alone, the staining of *E. faecalis* was improved with increasing concentrations, the geometric mean values of Van-Bodipy/Van fluorescence increased from 8.5 to 59.7 (Figure 1B). However, this strategy did not improve the staining for either *B. subtilis* or *E. faecalis* compared to Van-Bodipy alone. The staining procedure was therefore used as a mixture of Van-Bodipy/Van. It allowed to reduce the quantity of labeled vancomycin and preserve a staining efficacy for the same final concentration. At for 4 μg/mL, Van-Bodipy used alone or in combination with unlabeled vancomycin enabled to obtain geometric mean fluorescence values for the staining of *E. faecalis* of 34.5 and 38.5, respectively.

Increasing concentrations of WGA-647 from 10 to 100 μg/mL slightly improved the staining of Gram-positive bacteria (geometric mean values of WGA-647 fluorescence increased from 8.0 to 22.9 and from 250.0 to 351.0 for *B. subtilis* ATCC 23857 and *E. faecalis* JH2.2, respectively) but also resulted in an increase in staining of Gram-negative bacteria, for which geometric mean values of WGA-647 fluorescence increased from 1.0 to 1.5 (Figure 1C). The 20 μg/mL concentration was used for the rest of the experiments.

### Combination of Van-Bodipy/Van and WGA-647 With KCl Supplementation Better Discriminated Gram-Positive From Gram-Negative Bacteria

**FIGURE 2:**
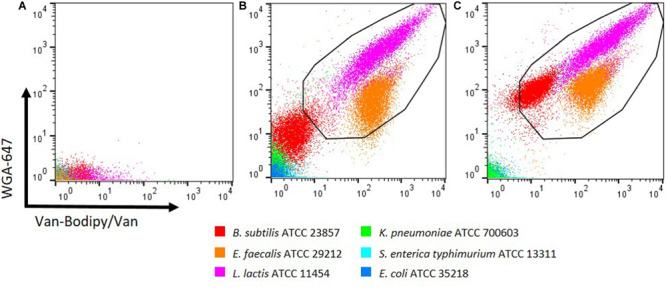
Optimization of the discrimination between Gram-positive and Gram-negative bacteria with KCl supplementation. Overnight culture of the tested bacteria were adjusted to an OD_600_ of 0.5. **(A)** Unstained bacterial species are represented. Staining was performed with the combination of Van-Bodipy/Van/WGA-647 in either presence of **(B)** PBS or **(C)** 1 M KCl in the dark at room temperature for 15 min. Gram-positive bacteria *B. subtilis* ATCC 23857, *E. faecalis* ATCC 29212, *L. lactis* ATCC 11454 are presented in red, orange and pink, respectively and Gram-negative bacteria *K. pneumoniae* ATCC 700603, *S. enterica typhimurium* ATCC 13311 and *E. coli* ATCC 35218 are presented in green, light blue and blue, respectively. Each bacterial species was stained separately and combined in one FCM plot.

Due to weak staining of *B. subtilis*, the discrimination between Gram-positive and Gram-negative bacteria was not optimal. The combination of both stains, Van-Bodipy/Van and WGA-647 was therefore investigated and unstained events are represented in Figure 2A. A better discrimination was obtained but for *B. subtilis* the fluorescence signal was still close to what was observed with Gram-negative bacteria (Figure 2B). As described by Holm and Jespersen (2003), the addition of 1 M KCl improved the staining efficacy, resulting in a clear distinction between *B. subtilis* and Gram-negative bacteria. Both staining by Van-Bodipy/Van as well as WGA-647 were improved for *B. subtilis* in the presence of 1 M KCl with an increase of the geometric mean fluorescence values of Van-Bodipy/Van and WGA-647 from 3.3 and 9.7 to 11.7 and 93.3, respectively (Figure 2C). A slight increase of WGA-647 staining was also observed for Gram-negative bacteria but the difference remained important compared to Gram-positive bacteria.

### PFA and Ethanol Fixation Methods Differently Affect the Optimized Staining Method

**FIGURE 3:**
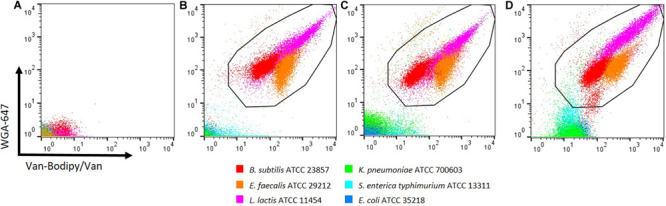
Effects of PFA and ethanol fixation methods on Gram staining. Overnight culture of the tested bacteria were adjusted to an OD_600_ of 0.5. **(A)** Unstained bacterial species are represented. Bacteria were stained in the dark at room temperature for 15 min with the combination of Van-Bodipy/Van/WGA-647 in the presence of 1 M KCl before fixation **(B)** or after 1 h fixation with 1% PFA **(C)** or 95% ethanol **(D)**. Gram-positive bacteria *B. subtilis* ATCC 23857, *E. faecalis* ATCC 29212, *L. lactis* ATCC 11454 are presented in red, orange, and pink, respectively and Gram-negative bacteria *K. pneumoniae* ATCC 700603, *S. enterica typhimurium* ATCC 13311 and *E. coli* ATCC 35218 are presented in green, light blue and blue, respectively. Each bacterial species was stained separately and combined in one FCM plot.

To determine whether the Gram staining method could be used after sample fixation, two methods: 1% PFA and 95% ethanol for 1 h were investigated. Unstained events and events stained in presence of 1 M KCl are represented in Figures 3A,B, respectively. PFA fixation did not modify much the staining efficacy of the tested Gram-positive bacteria (Figure 3C). Geometric mean values of Van-Bodipy/Van fluorescence slightly decreased from 62.3 to 41.7 and from 428.0 to 371.0 for *B. subtilis* and *L. lactis*, respectively, and geometric mean values of WGA-647 fluorescence decreased from 126.0 to 77.4 and from 455.0 to 320.0 for *B. subtilis* and *L. lactis*, respectively. These variations did not alter the discrimination between Gram-positive and Gram-negative bacteria. A slight increase of Gram-negative bacteria staining was noticed for which geometric mean values of Van-Bodipy/Van fluorescence increased from 1.3, 1.1 and 2.1 to 1.6, 1.9, and 3.3 for *E. coli*, *K. pneumoniae* and *S. enterica typhimurium*, respectively. Unlike PFA, fixation with 95% ethanol led to changes in staining, mostly for Gram-negative bacteria for which geometric mean values of Van-Bodipy/Van fluorescence increased from around 1.2 for all the Gram-negative bacteria to around 17.0 for *S. enterica typhimurium* and *K. pneumoniae* and around 22.0 for *E. coli* after ethanol fixation (Figure 3D). Discrimination between Gram-positive and Gram-negative bacteria was preserved but this fixation method should be used with caution as it can lead to misinterpretations when combined with Van-Bodipy/Van/WGA-647 staining.

### Different Strains of the Same Species Stain Differently With WGA-647

**FIGURE 4:**
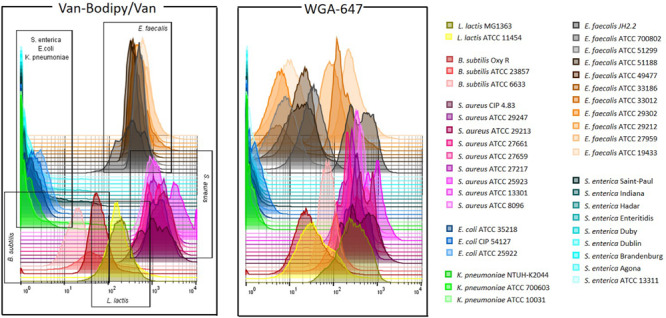
Gram staining flow cytometry analysis of aerobic/facultative anaerobic strains. Overnight cultures of the tested bacterial strains were adjusted to an OD_600_ of 0.5. Staining was performed with the defined combination of Van-Bodipy/Van/WGA-647 at their optimal concentrations of 2/2/20 μg/mL in 1 M KCl during 15 min at room temperature in the dark. Each strain was stained separately and combined in one FCM plot. The staining was performed on three biological replicates, but only one of the triplicate is represented in this figure.

The staining method combining Van-Bodipy/Van/WGA-647 was firstly validated using the 40 aerobic/facultative anaerobic strains belonging to *B. subtilis*, *E. faecalis*, *L. lactis*, *Staphylococcus aureus*, *E. coli*, *K. pneumoniae* and *S. enterica* species. The Van-Bodipy/Van staining was similar for all tested strains that belonged to a same species (Figure 4). Gram-negative bacteria were not or only slightly stained compared to Gram-positive bacteria. Geometric mean values of Van-Bodipy/Van and WGA fluorescence were not higher than 4.0 and 2.5, respectively, for all Gram-negative strains tested. Strains of *B. subtilis* were the most weakly stained among Gram-positive bacteria with geometric mean values of Van-Bodipy/Van fluorescence ranging from 18.0 to 70.0 depending on the strains. For *E. faecalis*, *S. aureus* and *L. lactis*, geometric mean values of Van-Bodipy/Van fluorescence were around 366.0, 1463.0, and 183.0, respectively. Unlike with Van-Bodipy/Van staining, strains responded differently to WGA-647 staining. Significant variability was observed within *E. faecalis* for which geometric mean values of WGA-647 fluorescence varied from 5.0 to 400.0, whereas little variations were observed for *S. aureus* (from 260.0 to 600.0 with an average around 389.0), *L. lactis* (from 40.0 to 240.0) and *B. subtilis* (from 29.0 to 100.0). Gram-positive bacteria could therefore be easily distinguished from Gram-negative bacteria with this staining.

### Testing of the Gram Staining Method on Several Strictly Anaerobic Commensal Strains

**FIGURE 5:**
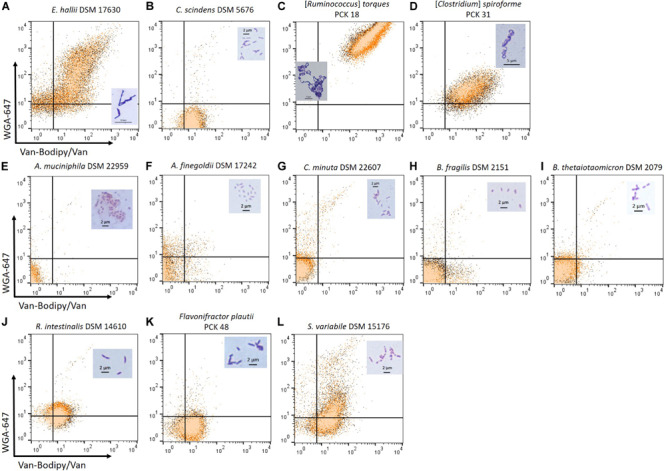
Van-Bodipy/Van/WGA-647 and Crystal violet Gram staining of Gram-positive, Gram-negative and Gram-variable, strictly anaerobic bacteria. The upper row corresponds to cultures of bacterial species described as Gram-positive including **(A)** *E. hallii* DSM 17630, **(B)** *C. scindens* DSM 5676 **(C)** [*Ruminococcus*] *torques* PCK 18 and **(D)** [*Clostridium*] *spiroforme* PCK 31. The middle row corresponds to bacterial species described as Gram-negative including **(E)** *Akkermansia muciniphila* DSM 22959, **(F)** *Alistipes finegoldii* DSM 17242, **(G)** *Christensenella minuta* DSM 22607, **(H)** *Bacteroides fragilis* DSM 2151 and **(I)** *Bacteroides thetaiotaomicron* DSM 2079. The lower row corresponds to bacterial species described as Gram-variable including **(J)** *R. intestinalis* DSM 14610, **(K)** *Flavonifractor plautii* PCK 48 and **(L)** *S. variabile* DSM 15176. All cultures were adjusted in the range of 10^5^ to 10^7^ events/mL based on flow cytometry quantification. Staining was performed with the defined combination of Van-Bodipy/Van/WGA-647 at their optimal concentrations of 2/2/20 μg/mL in 1 M KCl during 15 min at room temperature in the dark. Staining was performed on three biological replicates and each of them was combined in one FCM plot. In parallel, the same cultures were stained with crystal violet and safranin and the microscope slides were observed using the 100 X oil-immersion objective. Crystal violet staining of each bacterial species is represented on their respective FCM plots.

To determine if the combination of Van-Bodipy/Van/WGA-647 can be used on strictly anaerobic commensal strains, the staining was evaluated using a selection of species of interest. The staining was performed on three biological replicates for each strain (Figure 5). The upper, the middle and lower rows correspond to bacterial species usually described as Gram-positive, Gram-negative and Gram-variable bacteria, respectively. They are expected to be stained (Gram+) and unstained (Gram-) by the Van-Bodipy/Van/WGA-647 staining. The developed Van-Bodipy/Van/WGA-647 staining allowed identifying the tested species as Gram-positive bacteria except for *Clostridium scindens* DSM 5676 (Figure 5B) that was only slightly stained by Van-Bodipy/Van (geometric mean values of fluorescence of 12.9 ± 1.0) and almost not stained by WGA-647 (geometric mean values of fluorescence of 1.6 ± 0.1), which make it more difficult to differentiate from Gram-negative bacteria. A heterogeneous staining was observed for *Eubacterium hallii* DSM 17630 (Figure 5A). Separately from the Van-Bodipy/Van/WGA-647 staining, the Live/Dead staining was performed on the same culture. It allowed to correlate the population stained by Van-Bodipy/Van/WGA-647 that were associated with the “viable” fraction of *E. hallii* DSM 17630 culture. A similar effect occurred with *E. hallii* DSM 3353 and the evaluation at different time points highlighted variations in staining as a function of culture time (Supplementary Figure S2). All the fluorescent staining were in line with the crystal violet/safranin staining, with darker violet staining being obtained for both *E. hallii* strains (Figure 5A and Supplementary Figure S2) and [*Ruminococcus*] *torques* PCK 18 (Figure 5C) compared to *C. scindens* (Figure 5B). Among described Gram-variable bacteria, *Subdoligranulum variabile* can be classified as a Gram-positive bacterium based on the staining (Figure 5L). The fluorescence in the Van-Bodipy emission wavelength corresponded to the auto-fluorescence of the strain for which the geometric mean value was 11.3 ± 0.1. Fluorescence did not increase much with the Van-Bodipy/Van staining with a geometric mean value of 17.8 ± 0.5. No auto-fluorescence was observed in the WGA-647 emission wavelength. Both *R. intestinalis* (Figure 5J) and *Flavonifractor plautii* PCK 48 (Figure 5K) were slightly stained by Van-Bodipy/Van (geometric mean values of fluorescence of 10.5 ± 1.2 and 10.7 ± 1.1, respectively) and WGA-647 (geometric mean values of fluorescence of 9.1 ± 1.6 and 3.5 ± 0.4, respectively). All tested Gram-negative bacteria were not stained. The only exception was for *Prevotella copri*, the staining did not permit to identify it as a Gram-negative bacterium as fluorescence was observed in the Van-Bodipy emission wavelength, reaching geometric mean value of 12.5 ± 1.7 (data not shown).

### Evaluation of the Live/Dead Staining on Pure Cultures of Strictly Anaerobic Commensal Bacteria

**FIGURE 6:**
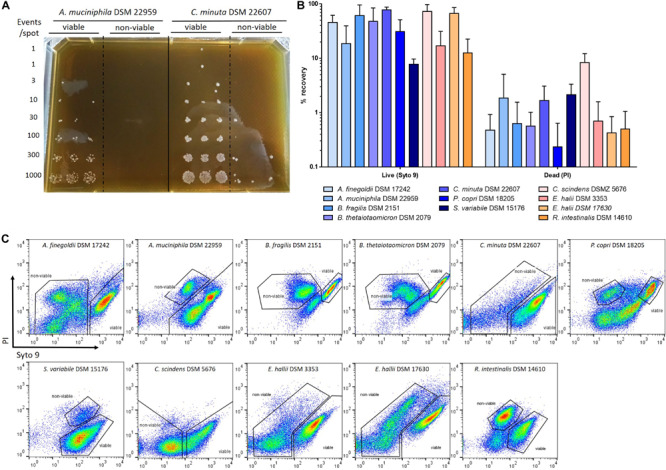
Culturability of the “viable”- and “non-viable”-stained fractions. Cultures of strictly anaerobic bacteria adjusted in the range of 10^7^ to 10^8^ events/mL based on flow cytometry quantification were stained with Syto 9 and PI and then sorted on mGAM plates. **(A)** Example of anaerobic sorting of “viable” and “non-viable”-stained fractions of *A. muciniphila* and *C. minuta*. **(B)** Percentage of recovery after sorting of “viable” and “non-viable”-stained fractions based on the Live/Dead staining. Percentages of recovery were calculated by reporting the number of observed colonies over the number of expected sorted events. Sorting experiments were performed on at least 3 biological replicates. **(C)** Representation of the gates used to performed the sorting of the different strictly anaerobic bacterial species.

In order to evaluate the efficacy of Live/Dead staining and its correlation with culturability, anaerobic sorting and culture of Syto 9- and PI-stained anaerobic bacteria was performed for several species of interest. Viabilities of cultures varied greatly depending on the considered strains (Supplementary Figure S3). The proportion of “viable” bacteria decreased to 40% after 2 days of culture for *C. scindens* and both *E. hallii* strains, whereas for *R. intestinalis* it took 3 days to reach this proportion (Supplementary Figure S3). For *B. fragilis*, *B. thetaiotaomicron* and *P. copri*, cultures rapidly reached a proportion of “viable” bacteria of less than 20% after 2 days (Supplementary Figure S3). Four to more than 7 days were required to observe significant mortality for other Gram-negative species, except for *C. minuta* for which approximatively 95% of bacteria were still labeled as “viable” after 10 days of culture (Supplementary Figure S3). Based on these viabilities, *P. copri*, both *E. hallii* strains, *B. thetaiotaomicron* and *B. fragilis* were harvested after 24 h of culture; *C. scindens* and *R. intestinalis* between 24 and 48 h and *A. muciniphila*, *C. minuta*, *A. finegoldii* and *S. variabile* after 5–7 days of culture. The sorting experiment was performed on three biological replicates for each strain. The percentage of recovery after sorting was calculated for each sorted “viable”- and “non-viable”-stained fractions based on the growth observed on culture plates (Figure 6A). The percentage of bacteria cultured from the “viable” fraction was greater than 10% for all species, ranging from 12% for *R. intestinalis* to 78% for *C. minuta*. The only exception was for *S. variabile*, for which less than 10% of the bacteria sorted and cultured from the “viable” fraction allowed the growth of colonies. Several colonies were still cultured from the “non-viable” fraction, corresponding to about 0.1–3% of the sorted bacteria. The only exception was for *C. scindens*, for which approx. 10% of bacteria sorted from the “non-viable” fraction were cultured (Figure 6B).

### Fermentation Experiment

To determine if the staining method could be used to follow evolution of complex ecosystems during fermentation processes, this method was evaluated on samples issued from batch fermentations of different fecal samples performed in different culture conditions. Triplicates of the three fecal samples F1, F2 and F3 were cultured in the Micro-24^®^ Microbioreactor in mGAM culture medium with a pH regulated at either 6.2 or 6.5. The dissolved oxygen concentrations were maintained at 0.01% in the entire cassette during the fermentation. The pH was difficult to regulate in our experiments, with a constant increase to reach 6.8 (F1 & F3) to 6.9 (F2) after 72 h of fermentation for the pH 6.5 condition, and 6.4 (F1 & F3) to 6.5 (F2) for the pH 6.2 condition (Supplementary Figure S4).

### Flow Cytometry as a First Analysis Level of Complex Ecosystems

**FIGURE 7:**
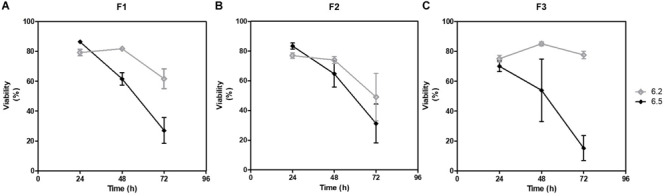
Evolution of estimated viabilities during fermentation. Viabilities of the cultured fecal samples were measured by flow cytometry along the 72 h of fermentation. At each sampling time, all samples were stained with Syto9 and PI. After 15 min incubation in the dark at room temperature, samples were analyzed in flow cytometry and viabilities were calculated for **(A)** F1, **(B)** F2, and **(C)** F3.

The compositions of the cultured fecal microbiota F1, F2 and F3 were analyzed by flow cytometry, which allowed to measure the number of bacteria and the ratio of PI/Syto 9 stained events approximating the overall viability. Gram-positive and Gram-negative proportions were also estimated. After 24 h of fermentation, the viabilities of the different cultured materials did not differ significantly, being around 70–85% for the three fecal samples, regardless of the initial pH condition. However, loss of viability was greater afterward, especially for pH 6.5 condition with less than 40% viability observed after 72 h of fermentation (Figure 7).

Bacterial biomass, as measured by FCM bacterial quantification, was enriched by 1.9 to 2.2 log units in the 5 mL cultures compared to the initial fecal samples after 48 h of fermentation. Gram staining with Van-Bodipy/Van/WGA-647 was also directly performed after sampling to follow-up the fermentation in terms of ecosystems enrichment. After 24 h of fermentation, FSC/SSC dot plots of unstained cultures showed no differences between triplicates for a given pH regulation but revealed a clear difference between cultures at pH 6.2 and 6.5 for the two fecal samples F1 and F3 (Supplementary Figures S5A, S7A). A higher number of groups was identified in pH 6.2 cultures, which tended to remain more similar to their respective baselines compared to pH 6.5 cultures. The Van-Bodipy/Van/WGA-647 staining confirmed the differences observed between pH 6.2 and 6.5 cultures for F1 and F3, with a higher number of groups positively stained by WGA-647, 1 and 2 groups, respectively, in pH 6.2 cultures compared to pH 6.5 cultures with only one or absence of groups positively stained by WGA-647 (Supplementary Figures S5B, S7B). The difference was less noticeable for the fecal sample F2 on both FSC/SSC and Van-Bodipy/Van/WGA-647 plots (Supplementary Figures S6A, S6B). As a result, unstained events were more represented in pH 6.5 cultures at 24 h of fermentation, with proportions of 68.5 ± 0.6% vs. 35 ± 1.9%, 63.7 ± 2.4% vs. 52.5 ± 1.2% and 81 ± 1.6% vs. 28.7 ± 1.4% for F1, F2 and F3, respectively. The bacterial communities did not change much between 24 and 48 h of fermentation (Supplementary Figures S5C,D, S6C,D, S7C,D).

### pH Regulation Modulates Fecal Microbiota Cultivation During Batch Fermentation

**FIGURE 8:**
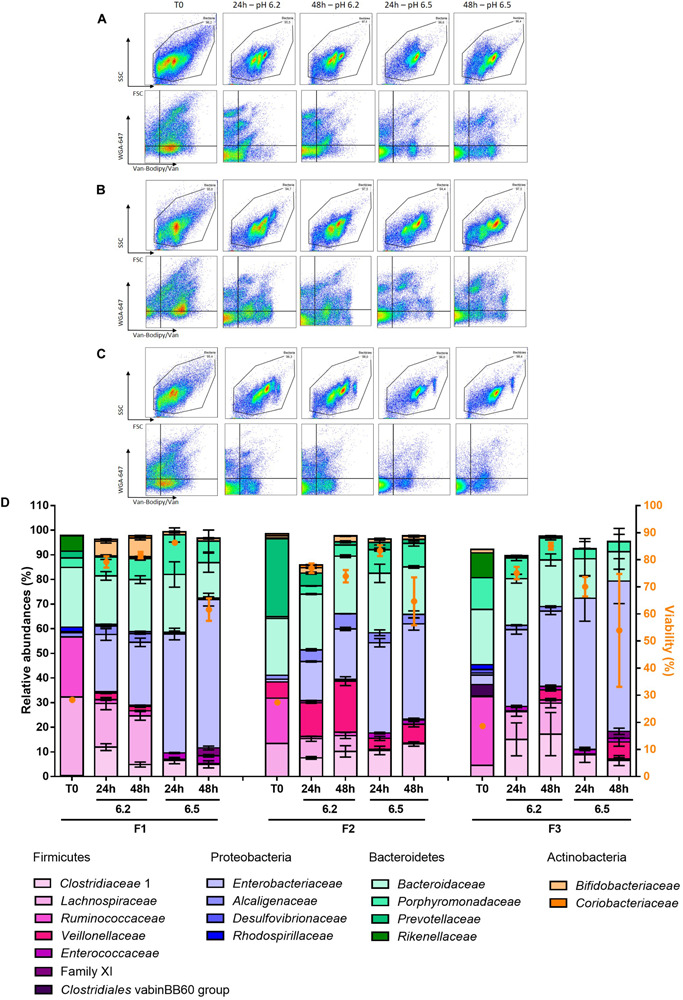
Relative abundances are represented at the family level of fecal samples F1, F2, and F3 after 24 and 48 h of fermentation associated with their cytometry analysis. Flow cytometry analysis are presented for **(A)** F1 fermentation, **(B)** F2 fermentation and **(C)** F3 fermentation. **(D)** 16S rRNA gene sequencing results are presented, only families with relative abundances greater than 1% in the baseline or after fermentation are represented. Data are represented as mean + SD of the triplicates except for the baselines.

The samples were then analyzed based on 16S rRNA gene sequencing and compared to their respective baselines. At the phylum and family levels, a lower pH regulation allowed a better preservation of the initial fecal microbiota composition. The four dominating phyla of the gut microbiota: Firmicutes, Bacteroidetes, Actinobacteria and Proteobacteria were represented in all the cultures. However, a dramatic amplification of Proteobacteria was observed, with the relative abundancies of Proteobacteria-related OTUs reaching 20–30% and 40–60% after 48 h of culture at pH 6.2 and pH 6.5, respectively (Figure 8 and Supplementary Table S2). After 24 h of fermentation and whatever the fecal sample, the *Clostridiaceae* 1 became the main family of the Firmicutes phylum, reaching relative abundances of about 60% (F1) and 50% (F3, F2) among Firmicutes for pH 6.5 cultures (Figure 8 and Supplementary Table S2). As a result, *Lachnospiraceae* and *Ruminococcaceae*, initially dominant, were considerably reduced in those cultures, reaching less than 1% of the Firmicutes. The *Lachnospiraceae* and *Ruminococcaceae* families were better preserved in pH 6.2 cultures but with important reduction of their relative abundances compared to the baselines (Figure 8 and Supplementary Table S2). Families of the Bacteroidetes phylum were relatively well preserved with the exception of the *Prevotellaceae* family for F2, whose abundance was drastically reduced whatever the pH regulation. The *Enterobacteriaceae* family dominated the Proteobacteria phylum and was approximatively two-fold lower in pH 6.2 cultures compared to pH 6.5 cultures (Figure 8 and Supplementary Table S2).

**FIGURE 9:**
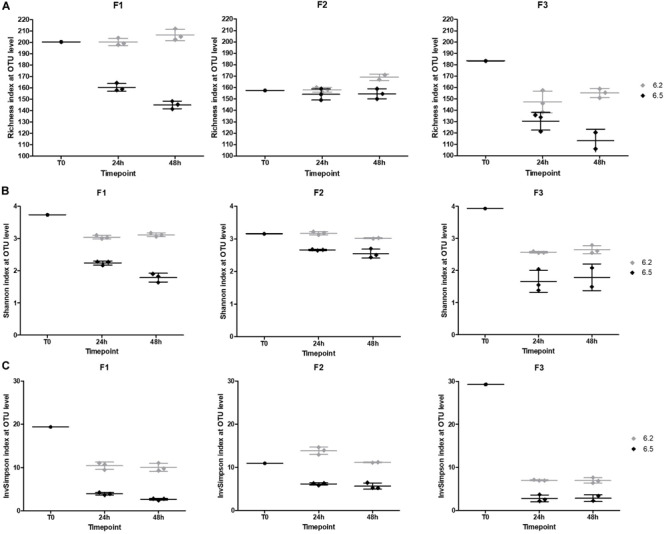
Obtained α-diversity indexes at the OTU level **(A)** Richness **(B)** Shannon index **(C)** Inverse Simpson index based on 16S rRNA gene sequencing.

We also looked at the α- and β-diversities of the cultured samples at the OTU level. Lower pH regulation resulted in similar richness to baselines, except for F3 (Figure 9A). The richness even surpassed the baselines after 48 h of fermentation. The Shannon and Inverse Simpson indexes displayed lower diversities in the cultures compared to initial fecal samples, except for F2 in pH 6.2 cultures after 24 h of fermentation (Figures 9B,C). Overall, alpha diversity was stable over time.

Even though the number of different taxa in the cultured fecal samples was mainly similar to their baseline, relative abundances could not be preserved and cultured fecal samples presented dissimilarities with their corresponding baseline (Supplementary Figure S8A). Greater similarities to the baselines, as measured with the Bray Curtis index at the genus level, were obtained in pH 6.2 cultures regardless of the initial fecal sample, around 30% for F1 and F3 and over 40% for F2 (Supplementary Figure S8B). The similarities were relatively stable over time.

### Comparison of Gram-Positive and Gram-Negative Bacterial Proportions Measured by FCM With Proportions Estimated From 16S rRNA Repertoire Sequencing

**FIGURE 10:**
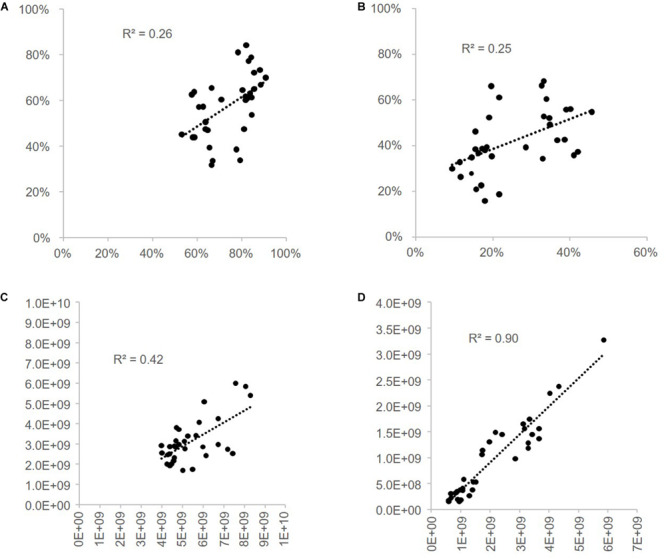
Correlation of Gram-positive and Gram-negative bacterial proportions estimated from 16S rRNA repertoire sequencing and flow cytometry. Gram-positive and Gram-negative bacterial proportions estimated by 16S rRNA gene sequencing using a relative or quantitative microbiome profiling (R- or QMP) approach were compared to flow cytometry proportions estimated with the Van-Bodipy/Van/WGA-647 staining method. Linear regression curves were obtained and R^2^-values were calculated: **(A)** correlation of Gram-negative bacteria RMP vs. FCM, **(B)** correlation of Gram-positive bacteria RMP vs. FCM, **(C)** correlation of Gram-negative bacteria QMP vs. FCM and **(D)** correlation of Gram-positive bacteria QMP vs. FCM.

Gram-positive and Gram-negative bacteria proportions measured by FCM were compared to the one calculated by 16S rRNA repertoire analysis using two different methods. R^2^-values calculated for the linear regression curves are presented in Figure 10. These values are 0.25 and 0.26 when the Gram-positive and Gram-negative proportions measured in cytometry are compared to the proportions deduced from the classical 16S rRNA analysis. They reach 0.42 (Gram-negative) and 0.90 (Gram-positive) when the proportions measured in flow cytometry are compared to proportions deduced from 16S rRNA data after a correction based on 16S rRNA gene-copy numbers and cell counts by sample (methods 2.12; Vandeputte et al., 2017).

## Discussion

Isolated strains as well as more or less complex mixtures of commensal strains are increasingly being explored as possible treatments for a large number of pathologies in both humans and animals. It is therefore becoming necessary to develop tools that make it possible to monitor the evolution of these ecosystems, whether it is in the context of R&D work aimed at exploring the influence of different parameters on the evolution of cultivated populations, or in an industrial context to monitor the production of strains of interest. High-throughput sequencing technologies offer a high level of precision to explore the phylogenetic composition of microbial populations, but suffer from certain limitations: lack of measurement of bacterial viability, measurement of the relative rather than absolute abundance of populations, but above all still relatively high cost and significant delays in delivering sequencing results.

In this context, FCM adapted to bacterial cell represents an interesting, fast and cost-effective technology for the rapid assessment of complex microbial ecosystems dynamics and overall viability of their constituents. Compared to eukaryotic cells analysis, bacterial cells analysis using FCM is still limited by the relatively low number of fluorescent stains available. Nucleic acid stains are mainly used as well as lectins, labeled antibiotics or specific dyes allowing to evaluate membrane potential or metabolic activities (Muller and Nebe-von-Caron, 2010). In this study, we proposed to evaluate the use of the classical Live/Dead staining method as well as the combination of labeled WGA and vancomycin as external structural stains to identify and discriminate Gram-positive from Gram-negative bacteria in complex ecosystems. Both staining methods were first evaluated with a series of isolated strains, including strict anaerobes, and were then used to follow the evolution of complex microbiota in fermentation.

The use of 4 μg/mL of Van-Bodipy/Van in combination with 20 μg/mL of WGA-647 in 1 M KCl was determined to be the optimal staining condition to discriminate Gram-positive from Gram-negative bacteria. As previously described, the supplementation of 1 M KCl improved the staining of some Gram-positive bacteria by both WGA-647 and Van-Bodipy/Van (Holm and Jespersen, 2003). KCl can destabilize external structures, including the teichoic acids, which are normally rigid in water and change to a random coil configuration in the presence of salt (Doyle et al., 1974), thus leading to a greater accessibility of the patterns recognized by Van-Bodipy/Van and WGA-647. The 1% PFA fixation can possibly be used to postpone analysis of samples as the discrimination of Gram-positive from Gram-negative bacteria was not altered. Conversely, ethanol may have induced the dissolution of membrane lipids, leading to the modification of proteins tertiary structures (Hobro and Smith, 2017) and unmasking the terminal D-Ala-D-Ala chains of the peptidoglycan that were previously not accessible, resulting in increased staining of Gram-negative bacteria. The Van-Bodipy/Van/WGA-647 staining screening was effective to discriminate different Gram-positive bacterial species or even strains as observed for *E. faecalis*. Bacteria that belong to this species exhibit capsular polysaccharides (Hancock and Gilmore, 2002) defining different serotypes based on their diversity (Hufnagel et al., 2004; Thurlow et al., 2009). Being differently expressed among strains of *E. faecalis*, such capsules can mask the recognition structures of the WGA and prevent its binding to the peptidoglycan. Only weak staining by both Van-Bodipy/Van and WGA-647 was observed for commensal anaerobic species *R. intestinalis*, *F. plautii* (PCK48) and *C. scindens*. This was not completely unexpected for *F. plautii* and *R. intestinalis* that were originally described as Gram-variable species (Duncan, 2002; Carlier et al., 2010). *C. scindens* is described as a Gram-positive species but it has the ability to form spores, which might impact staining efficacy. The Van-Bodipy/Van/WGA-647 staining of *S. variabile* classifying it as a Gram-positive bacterium is inconsistent with the literature in which *S. variabile* is described as a Gram-negative stained bacterium. However, the staining with the commercial LIVE BacLight^TM^ Bacterial Gram Stain Kit (Thermofisher) combining Syto 9 and HI, the latter preferably labeling Gram-positive bacteria, also stained *S. variabile* as a Gram-positive bacteria (Supplementary Figure S9). The staining with HI confirms the classification of *S. variabile* as a monoderm Firmicutes (Megrian et al., 2020). In addition, the strain was sensitive to vancomycin with a minimal inhibitory concentration of 4 mg/L, and contains in its genome conserved sequences coding for Leucine-Proline-x-Threonine-Glycine (LPxTG) motifs surface proteins anchored to the peptidoglycan (reference sequences WP_147644604.1 and WP_007048259.1) in Gram-positive bacteria, thus strengthening the previous results. Finally, in our experiments the Van-Bodipy/Van/WGA-647 staining was optimized using culture harvested at the end of the log-growth phase. It might be interesting to look at potential staining variations as function of growth stage of bacterial pure cultures (Beveridge, 1990).

The Live/Dead^®^ BacLight^TM^ staining has already been used by others to evaluate the proportions of “viable” and “non-viable” bacteria in fecal microbiota using flow cytometry, with a relatively good congruence between culturability and FCM results when samples are protected from oxygen exposure (Bellali et al., 2019; Burz et al., 2019). However, it has also been reported that PI staining that is included in the kit can be misleading for specific bacterial species, especially when bacteria are collected during early exponential growth and have high membrane potential (Shi et al., 2007; Kirchhoff and Cypionka, 2017). Due to these limitations we were interested to evaluate the culturability of fractions detected as “viable” or “non-viable” with the Live/Dead^®^ BacLight^TM^ staining kit for several, strictly anaerobic species of interest. In our study, up to four populations with different levels of Syto 9 and PI staining were observed depending on the tested anaerobic species (Figure 6C). These results are not surprising since intermediate states characterized by different levels of Syto 9 and PI staining of the cells have already been described by others (Berney et al., 2007). Anaerobic sorting of the “viable” fractions allowed the culture of more than 10% of the sorted events. The only exception was for *S. variabile* for which auto-fluorescence in the Syto 9 emission wavelength could have possibly biased the analysis. Misleading results were also obtained for *C. scindens* for which significant growth was observed for the sorted fraction identified as “non-viable.” *C. scindens* presented an uncommon staining pattern, with a weak Syto 9 and a weak PI staining (Figure 6C). The ability of *C. scindens* to form spores could possibly be a reason for these staining patterns, with bacterial DNA being more difficult to access due to the spore coat (Magge et al., 2009). It is worth noting that PI can potentially be toxic for bacteria and could have biased the results obtained for the PI-stained sorted events. Viability analysis should therefore be taken with care when applied on complex ecosystems, as they are represented by a wide range of bacterial species that can potentially respond differently to PI staining.

The dramatic expansion of Proteobacteria during the batch fermentation experiments has already been reported by others (Takagi et al., 2016; Sasaki et al., 2017; O’Donnell et al., 2018; Wiese et al., 2018). Higher relative abundances of Proteobacteria were obtained after 24 h of fermentation in our study compared to batch fermentation experiments performed which the same mGAM culture medium and in the same pH conditions (Takagi et al., 2016). The more important amplification of *Escherichia* observed in pH 6.5 compared to pH 6.2 cultures is consistent with the study of Ilhan et al. (2017) which showed a predominance of this genus in cultures started at pH 6.5 and 6.9. Fast-growing, facultative anaerobic bacterial species that belong to this genus can easily gain an advantage over slow-growing, obligate anaerobes during initial stages of fermentation, which can explain rapid expansion of an initially low-abundance population. The pH regulation was a key factor affecting the recovery of bacterial families and genera, which were always better preserved under culture conditions with a pH regulated at 6.2, which is consistent with the study of Walker et al. (2005) in which a better preservation of genera of interest was obtained with a pH regulated at 5.5 compared to 6.7. The three fecal sample used in the experiment evolved differently during the fermentation, resulting in differences in cultured families or genera. The influence of the initial complex microbial community was previously demonstrated in batch fermentation cultures (Perrotta et al., 2017; Van den Abbeele et al., 2018). The fermentation led to dissimilarities between the cultured samples and baselines, a phenomenon that was previously observed in a comparable 24-well *in vitro* fermentation system (O’Donnell et al., 2018).

The optimized Van-Bodipy/Van/WGA-647 staining method represents a first level of analysis of complex microbial ecosystems obtained by *in vitro* fermentation. This staining method revealed strong similarities between population communities obtained for triplicates, as well as the dramatic influence of the pH conditions on the culture of bacterial communities, which was further confirmed by 16S rRNA gene sequencing (Supplementary Figures S5, S6, and S7). In a previous study, FCM was used to quantitatively recalculate relative abundances obtained by 16S rRNA gene sequencing based on bacterial counts (Vandeputte et al., 2017). Instead of rarefying output to equal number of reads for each sample, Vandeputte et al. (2017) transformed the sequencing relative abundances data into quantitative microbiome profiles using sample cell counts. Interestingly, we obtained better correlation values with FCM measurements when this rather than the classical relative abundancy approach was used to estimate Gram proportions based on OTU Gram assignment. This was especially true for Gram-positive bacteria. This observation must be tempered by the fact that these analyses remain potentially highly biased. As observed on pure cultures, the Gram status is sometimes difficult to determine for specific bacterial species/strains, and it is risky to assign a Gram status to an OTU potentially corresponding to several species, some of which have never been cultivated and whose Gram status is therefore not known. The value of the number of 16S rRNA operons used as a corrective factor in the quantitative microbiome profiling approach is also highly susceptible to bias since this value is not known for many species and may also vary between strains (Louca et al., 2018). In this context, an interesting analysis would be to sort sub-communities according to their Gram status measured using FCM and then sequence 16S rRNA gene repertoire of the sorted fractions, which is planned in future experiments.

## Conclusion

Our study describes a rapid and cost-effective FCM-based tool using labeled WGA and vancomycin that can be used to rapidly generate compositional population community patterns of complex microbial ecosystems obtained by *in vitro* fermentations. It is potentially useful as a cost-effective tool allowing to follow changes throughout fermentation processes, with the goal to select samples that should be further analyzed using sequencing. This combination of staining enabled to give general trends on the evolution of a given sample. We also observed that the Live/Dead^®^ BacLight^TM^ viability kit was a reliable tool to assess the viability of strictly anaerobic commensal species tested in our study, with the notable exception of two species for which auto-fluorescence and sporulation might have biased the analysis. These biases should be kept in mind when analyzing complex microbial communities. In the future, FCM used in conjunction with the Van-Bodipy/Van/WGA-647 staining could represent a first analytical tool to qualify ecosystems presenting high levels of complexity, including commensal as well as environmental samples. The staining method would likely be improved if used in combination with other staining such as the DNA intercalating dye DAPI, that was shown to reveal heterogeneity and dynamics changes of intestinal microbiota (Zimmermann et al., 2016) but which requires the use of a UV laser. FCM can also be used to qualify single-strain probiotics or complex mixtures of bacteria resulting from industrial fermentations. Finally, it can be assumed that FCM would likely be useful to follow the evolution of well-defined microbiota in pre-clinical animal models such as gnotobiotic mice models (Brugiroux et al., 2016).

## Data Availability Statement

The datasets generated for this study can be found in the SRA/PRJNA609248/https://www.ncbi.nlm.nih.gov/sra/PRJNA609248, FlowRepository/FR-FCM-Z2H9/https://flowrepository.org.

## Author Contributions

AD, SB, CS, JD, and VT conceptualized the experiments. AD performed all the staining and fermentation experiments. SB contributed to the staining experiments. CG performed the analysis and assisted in the interpretation of sequencing data. AD and VT wrote the manuscript. All authors read and approved the final manuscript.

## Conflict of Interest

AD, CG, and CS are employed by the company MaaT Pharma.

The remaining authors declare that the research was conducted in the absence of any commercial or financial relationships that could be construed as a potential conflict of interest.

## Footnotes

1 http://www.R-project.org
