# Supplementary material for: Assessment of Gram- and Viability-Staining Methods for Quantifying Bacterial Community Dynamics Using Flow Cytometry
Source: Front Microbiol. 2020 Jun 26;11:1469. doi: 10.3389/fmicb.2020.01469 (PMC7333439; doi:10.3389/fmicb.2020.01469)
Supplement: Supplementary file 2 [file Data_Sheet_2.docx]

Supplementary Material

# Supplementary Figures

**
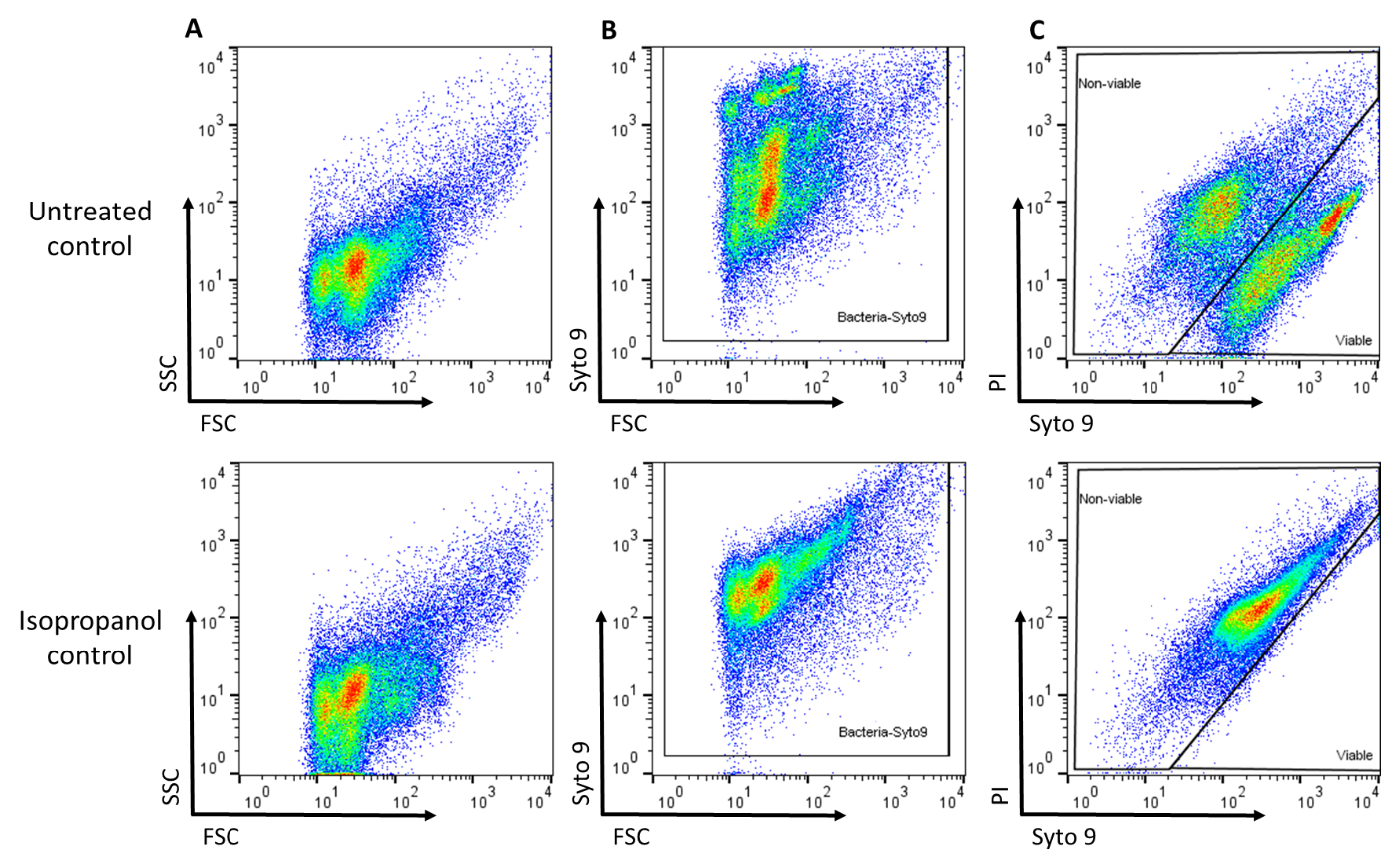
**

**Supplementary Figure S1** Gating strategy for the viability analysis. A reference fecal sample untreated or treated with isopropanol was stained with Syto 9 and PI and gates were defined for viability analysis. **(A)** Example of FSC/SSC plot of a reference fecal sample untreated or treated with isopropanol, **(B)** All nucleic acid stained by Syto 9 were gated as “Bacteria­‑Syto 9” and further selected for the analysis **(C)** The selected population “Bacteria‑Syto 9” was then analyzed by choosing fluorescence collected through 540/30 nm and 670/30 nm BP filters for Syto 9 and PI respectively, to determine “viable” and “non‑viable” fractions.

**
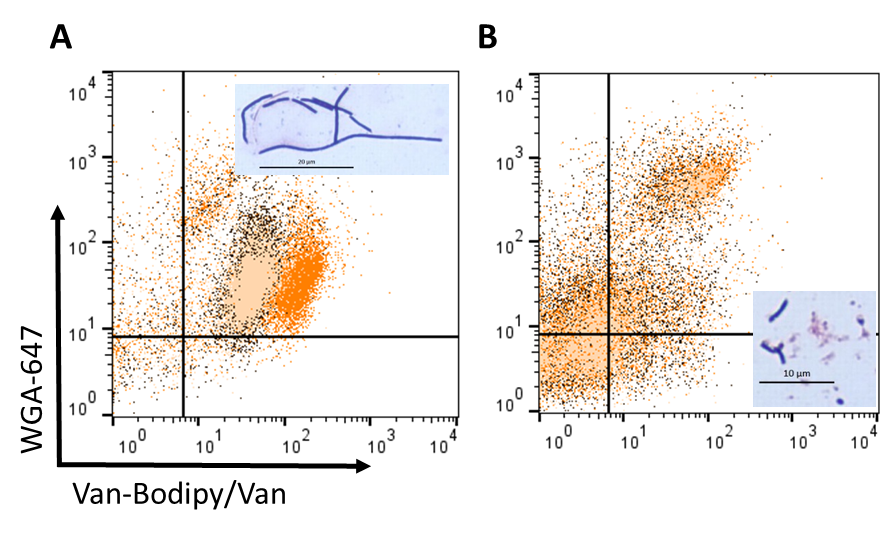
**

**Supplementary Figure S2** Flow cytometry and Crystal violet Gram staining of Gram-positive strictly anaerobic bacteria. **(A)** *E. hallii* DSM 3353, 6 h of culture, **(B)** *E. hallii* DSM 3353, 24 h of culture. Cultures were adjusted in the range of 10^5^ to 10^7^ events/mL based on flow cytometry quantification. Staining was performed with the defined combination of Van-Bodipy/Van/WGA-647 at their optimal concentrations of 2/2/20 µg/mL in 1 M KCl during 15 min at room temperature in the dark. Staining was performed on three biological replicates and each of them was combined in one FCM plot. In parallel, the same cultures were stained with crystal violet and safranin and the microscope slides were observed using the 100 X oil-immersion objective.

**
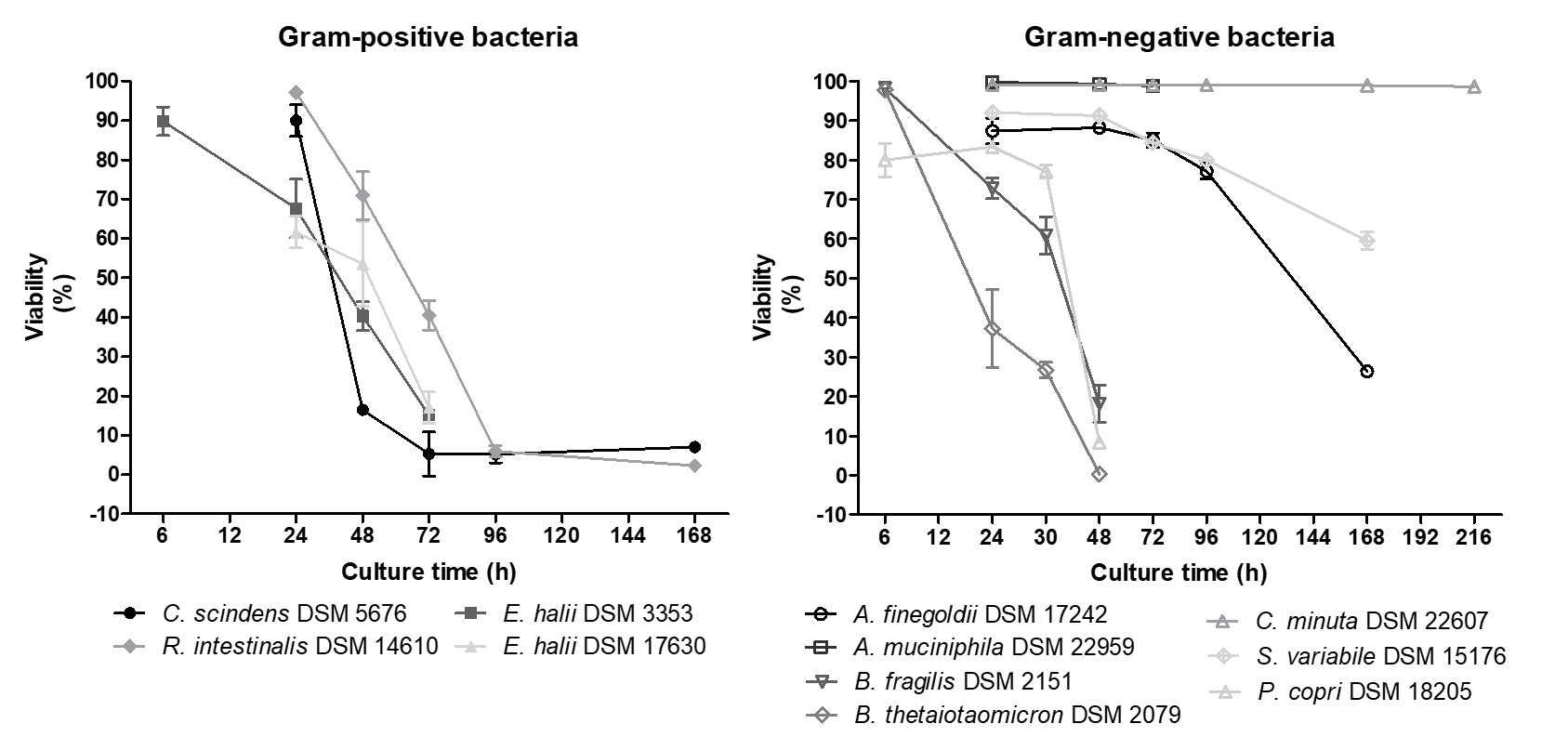
**

**Supplementary Figure S3** Gram-positive and Gram-negative bacteria viabilities over time. At each sampling time, 100 µL of each culture was collected to evaluate the viability with Syto 9 and PI using flow cytometry.

**
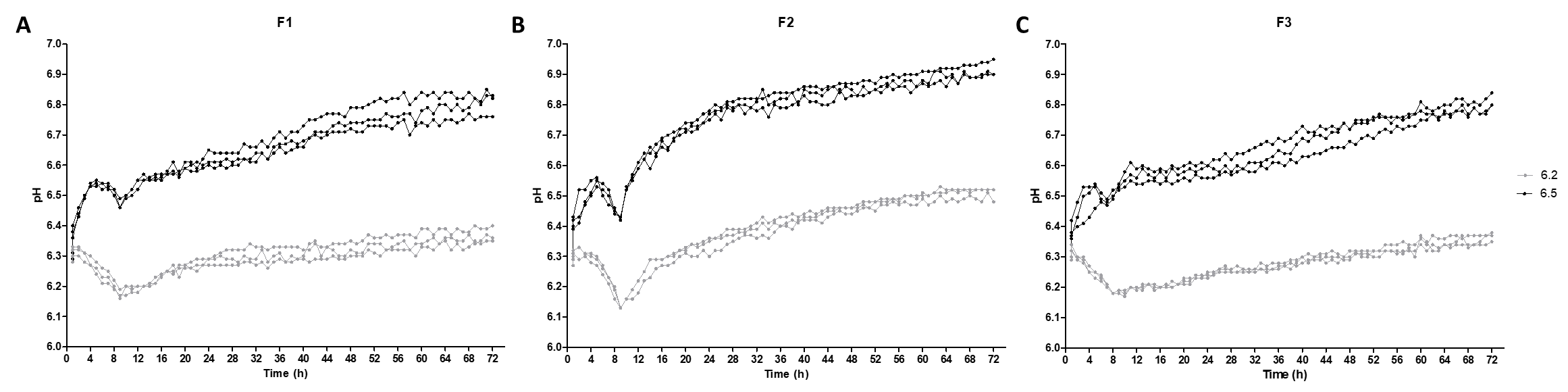
**

**Supplementary Figure S4** pH evolution of **(A)** F1, **(B)** F2 and **(C)** F3 cultures over the 72 h fermentation period


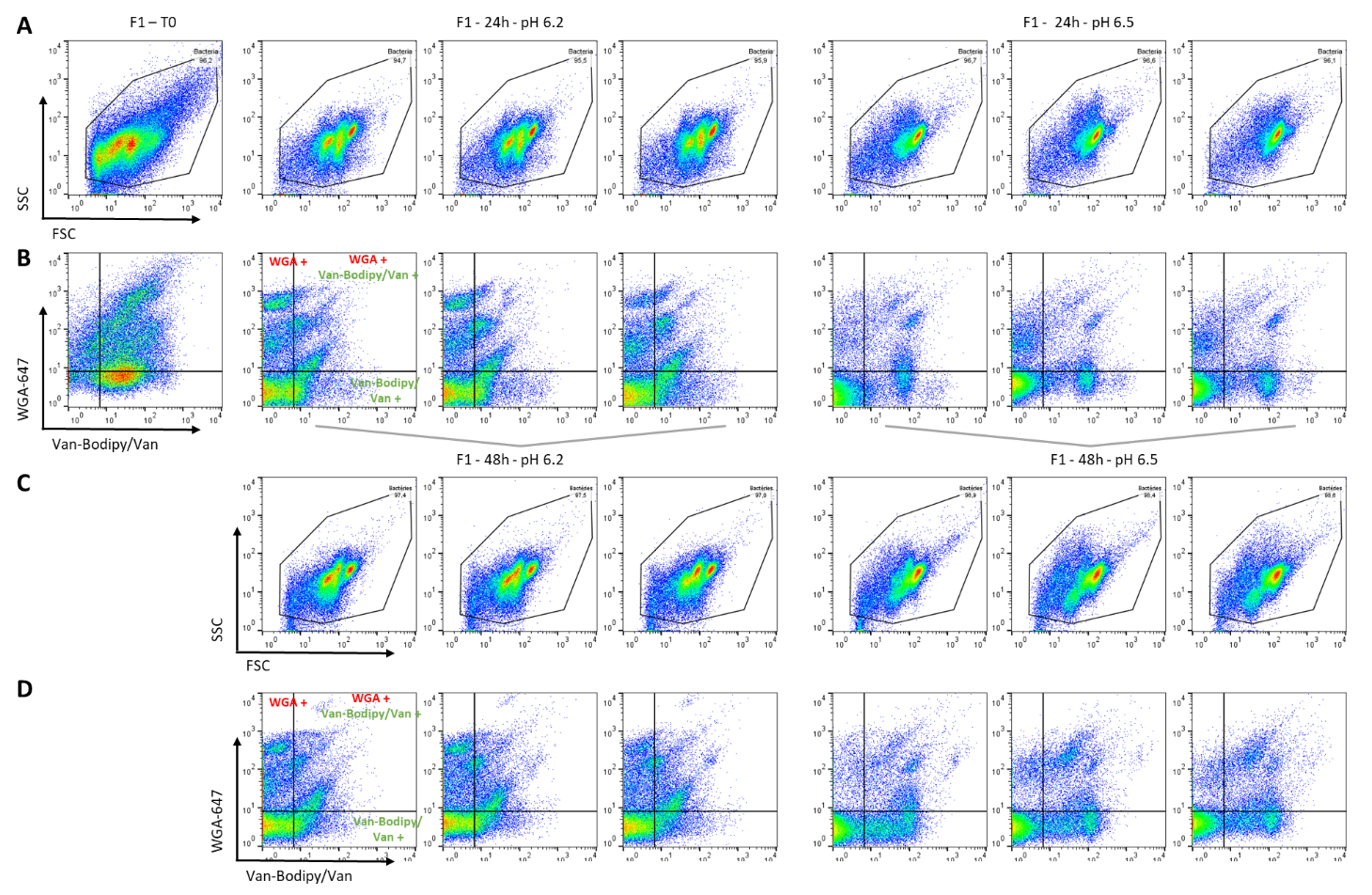


**Supplementary Figure S5** FSC/SSC and Van-Bodipy/Van/WGA-647 staining plots after 24 h and 48 h of F1 fermentation. **(A)** FSC/SSC plots at 24 h, **(B)** Van-Bodipy/Van/WGA-647 plots at 24 h, **(C)** FSC/SSC plots at 48 h and **(D)** Van-Bodipy/Van/WGA-647 plots at 48 h. Staining was performed with the defined combination of Van-Bodipy/Van/WGA-647 at their optimal concentrations of 2/2/20 µg/mL in 1 M KCl during 15 min at room temperature in the dark.

**
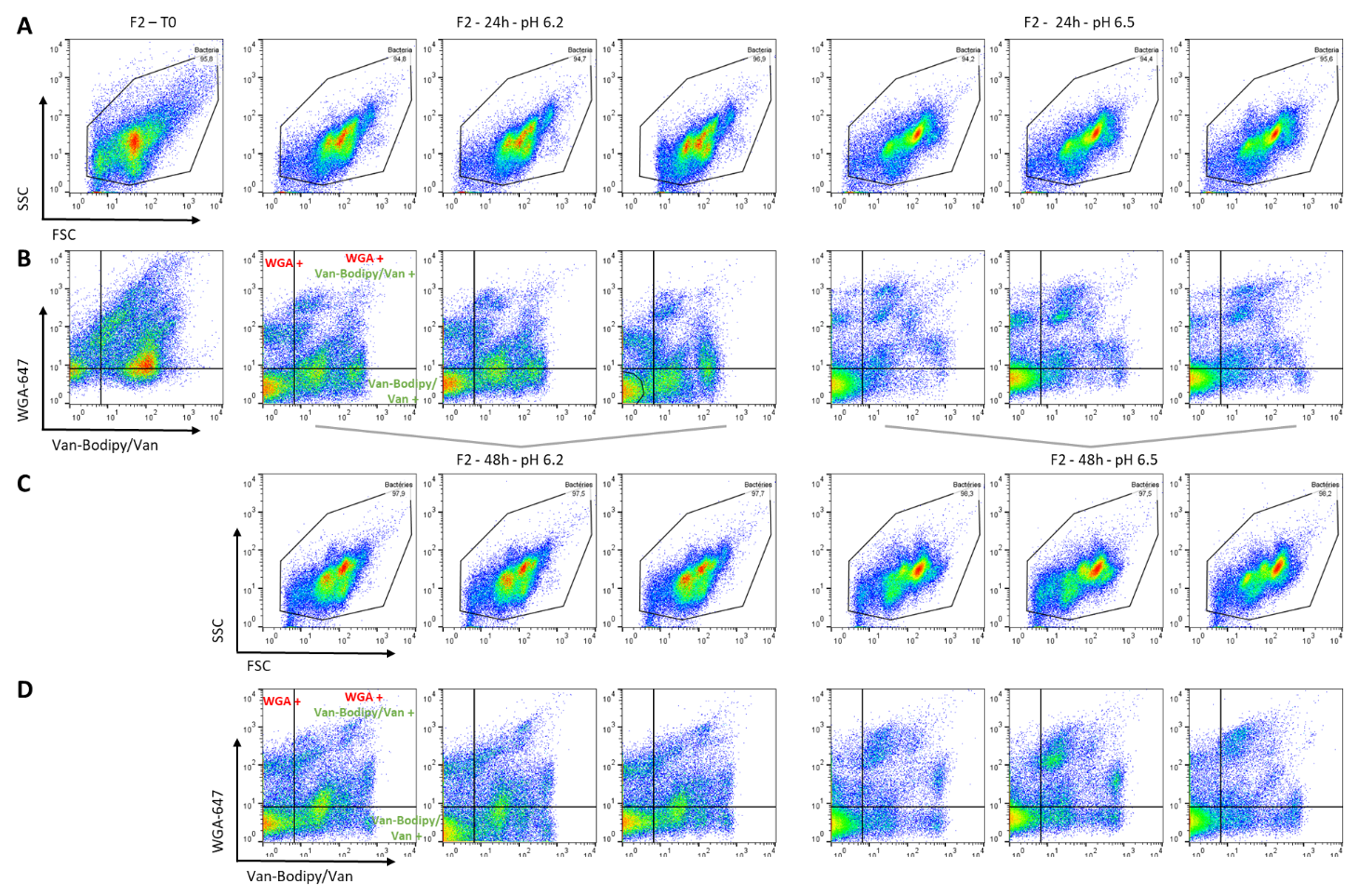
**

**Supplementary Figure S6** FSC/SSC and Van-Bodipy/Van/WGA-647 staining plots after 24 h and 48 h of F2 fermentation. **(A)** FSC/SSC plots at 24 h, **(B)** Van-Bodipy/Van/WGA-647 plots at 24 h, **(C)** FSC/SSC plots at 48 h and **(D)** Van-Bodipy/Van/WGA-647 plots at 48 h. Staining was performed with the defined combination of Van-Bodipy/Van/WGA-647 at their optimal concentrations of 2/2/20 µg/mL in 1 M KCl during 15 min at room temperature in the dark.

**
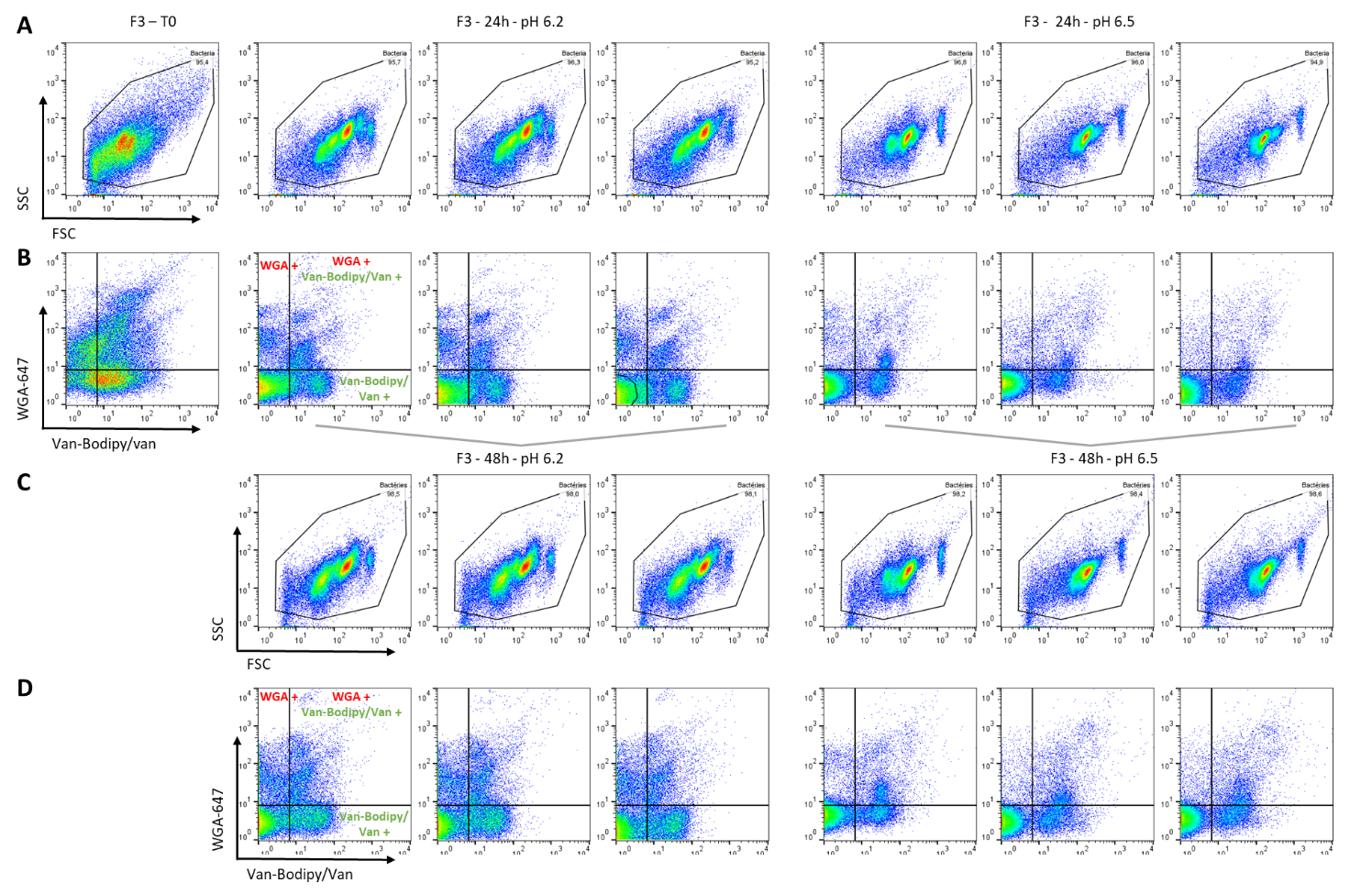
**

**Supplementary Figure S7** FSC/SSC and Van-Bodipy/Van/WGA-647 staining plots after 24 h and 48 h of F3 fermentation. **(A)** FSC/SSC plots at 24 h, **(B)** Van-Bodipy/Van/WGA-647 plots at 24 h, **(C)** FSC/SSC plots at 48 h and **(D)** Van-Bodipy/Van/WGA-647 plots at 48 h. Staining was performed with the defined combination of Van-Bodipy/Van/WGA-647 at their optimal concentrations of 2/2/20 µg/mL in 1 M KCl during 15 min at room temperature in the dark.


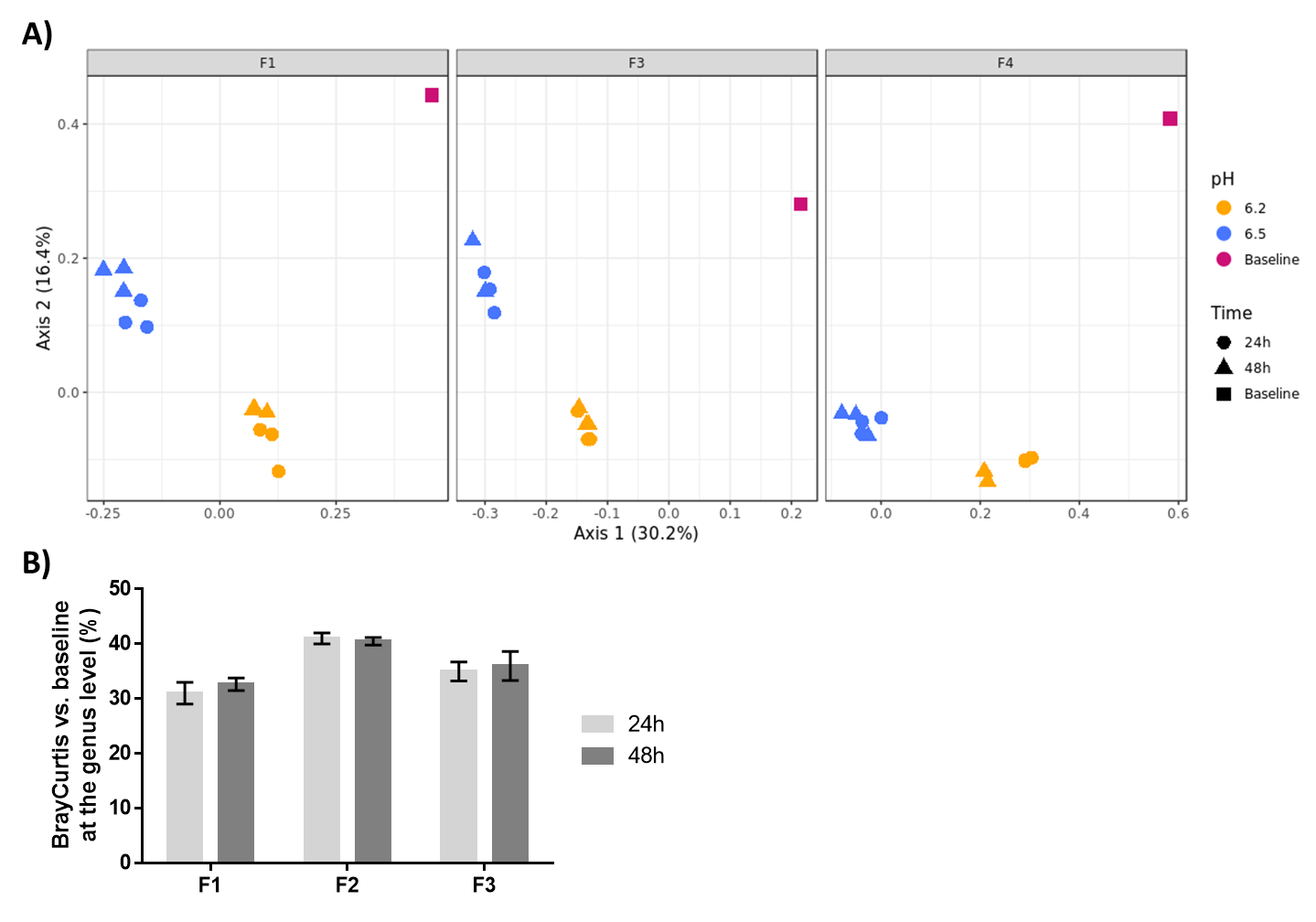


**Supplementary Figure S8 Bray‑Curtis similarity measures. A)** PCoA based on the Bray-Curtis dissimilarity measure and B) Bray‑Curtis similarity at the genus level, for F1, F2 and F3 after 24 h and 48 h of fermentation.

**
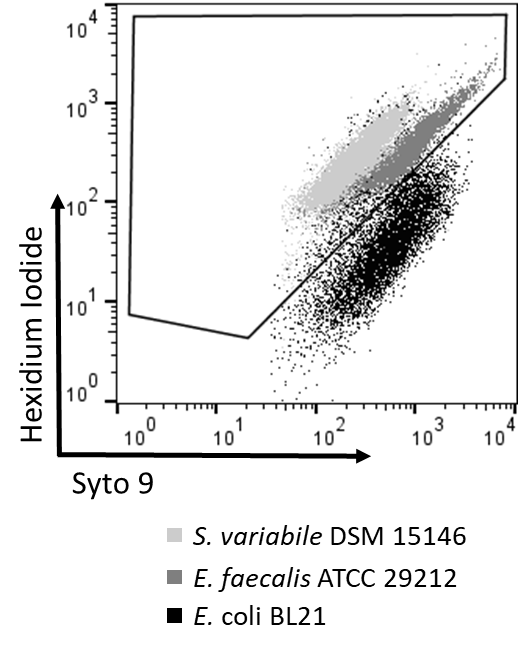
**

**Supplementary Figure S9** Evaluation of the commercial LIVE BacLight^TM^ Bacterial Gram Stain Kit. Overnight culture of *E. coli* BL21, *E. faecalis* ATCC 29212 and *S. variabile* DSM 15146 were adjusted in the range of 10^5^ to 10^7^ events/mL based on flow cytometry quantification and staining was performed as recommended by the manufacturer. Each species was stained separately and combined in the plot for analysis.

# Supplementary table

**Supplementary Table S1**. Quantitative microbiome profiling at the genus level

**Supplementary Table S2.** Relative abundances obtained by 16S rDNA sequencing represented at the phylum and family levels of fecal samples F1, F2 and F3 after 24 h and 48 h of fermentation.
